# A cardiac contouring atlas for radiotherapy

**DOI:** 10.1016/j.radonc.2017.01.008

**Published:** 2017-03

**Authors:** Frances Duane, Marianne C. Aznar, Freddie Bartlett, David J. Cutter, Sarah C. Darby, Reshma Jagsi, Ebbe L. Lorenzen, Orla McArdle, Paul McGale, Saul Myerson, Kazem Rahimi, Sindu Vivekanandan, Samantha Warren, Carolyn W. Taylor

**Affiliations:** a Clinical Trial Service Unit, Nuffield Department of Population Health, University of Oxford, UK; b Medical Research Council Population Health Research Unit, Nuffield Department of Population Health, University of Oxford, UK; c Department of Oncology and Haematology, Queen Alexandra Hospital, Portsmouth, UK; d Department of Radiation Oncology, University of Michigan, Ann Arbor, USA; e Laboratory of Radiation Physics, Odense University Hospital, Denmark; f St. Luke's Radiation Oncology Network, Dublin, Ireland; g Division of Cardiovascular Medicine, Radcliffe Department of Medicine, University of Oxford, UK; h George Institute for Global Health, University of Oxford, UK; i CRUK/MRC Oxford Institute for Radiation Oncology, Gray Laboratories, University of Oxford, UK; j University of Birmingham NHS Foundation Trust, Birmingham, UK

**Keywords:** Contouring, Cardiac structures, Radiotherapy CT-planning scans, Atlas

## Abstract

**Background and purpose:**

The heart is a complex anatomical organ and contouring the cardiac substructures is challenging. This study presents a reproducible method for contouring left ventricular and coronary arterial segments on radiotherapy CT-planning scans.

**Material and methods:**

Segments were defined from cardiology models and agreed by two cardiologists. Reference atlas contours were delineated and written guidelines prepared. Six radiation oncologists tested the atlas. Spatial variation was assessed using the DICE similarity coefficient (DSC) and the directed Hausdorff average distance (${\overset{\to }{d}}_{H\text{,}\mathrm{avg}}$). The effect of spatial variation on doses was assessed using six different breast cancer regimens.

**Results:**

The atlas enabled contouring of 15 cardiac segments. Inter-observer contour overlap (mean DSC) was 0.60–0.73 for five left ventricular segments and 0.10–0.53 for ten coronary arterial segments. Inter-observer contour separation (mean ${\overset{\to }{d}}_{H\text{,}\mathrm{avg}}$) was 1.5–2.2 mm for left ventricular segments and 1.3–5.1 mm for coronary artery segments. This spatial variation resulted in <1 Gy dose variation for most regimens and segments, but 1.2–21.8 Gy variation for segments close to a field edge.

**Conclusions:**

This cardiac atlas enables reproducible contouring of segments of the left ventricle and main coronary arteries to facilitate future studies relating cardiac radiation doses to clinical outcomes.

Radiotherapy increases cure rates in breast cancer, Hodgkin lymphoma, oesophageal cancer and lung cancer. However, in each of these cancers, radiotherapy may involve some cardiac exposure, thereby increasing the risk of several different heart diseases. Oncologists need to know the relationships between cardiac doses and various types of heart disease so they can estimate radiation-related risks for patients. A number of studies have derived dose–response relationships estimating the risk of various cardiac outcomes in terms of whole heart doses. One study of women irradiated for breast cancer, based on around 1,000 events, showed that the risk of a major coronary event increased by 7.4% per Gy mean heart dose [1]. Several smaller studies related whole heart dose to cardiac death, valvular heart disease, decreased myocardial function and pericardial disease [2], [3].

Whole heart dose may not be the best predictor of all types of radiation-related heart disease. Some studies have investigated the relationship between doses to particular cardiac substructures and subsequent damage to those structures. In Hodgkin lymphoma, one study related valve doses to subsequent valvular heart disease and another related coronary artery doses to subsequent coronary artery stenosis [4], [5]. In breast cancer, two studies have related coronary artery doses to subsequent coronary artery stenosis [6], [7] and further studies have related whole left ventricle dose to subsequent subclinical left ventricular abnormalities [8], [9]. But the relationships between doses to different left ventricular or coronary artery segments and subsequent injury of these segments have not yet been investigated.

The heart is a complex anatomical organ made up of muscle, arteries and valves and it can be difficult to contour cardiac substructures reproducibly. In 2010, the Quantitative Analyses of Normal Tissue Effects in the Clinic (QUANTEC) Report highlighted the need for guidelines to reduce inter-observer variation in cardiac contouring [2]. In 2011, investigators at the University of Michigan developed a cardiac atlas describing contouring of the cardiac chambers, coronary arteries, conduction system and valves [10]. We now present an atlas for contouring different segments of the coronary arteries and the left ventricle on radiotherapy planning CT scans.

## Material and methods

### Atlas development

Information was sought from (1) anatomy [11] and cardiac imaging [12] textbooks (2) IMAIOS e-anatomy [13] (3) key articles describing cardiac segmentation models or normal cardiac anatomy [14], [15], [16], [17], [18], [19] and (4) the echocardiogram and angiogram reports of 500 women included as cases in a population-based study of women who had a major coronary event after breast cancer radiotherapy [1]. These reports verified that the segments in the atlas matched those described by cardiologists regarding the location of damage so that segment doses may be directly related to location of injury in future dose–response relationships. These four sources were used to define five left ventricular segments (Table 1, Supplementary Fig. 1) and ten coronary artery segments (Table 2). We ensured that each contour definition was reproducibly identifiable on non-contrast radiotherapy CT-planning scans and large enough to allow accurate dose calculation.

### Selection of atlas CT set

Ten radiotherapy CT-planning scans were randomly selected from women irradiated for left-sided breast cancer at Odense University Hospital in the year 2010. The treatment position was supine with both arms above the head. CT scan slice thickness was 3 mm and intravenous contrast was not used. All segments were contoured by a radiation oncologist (FD) on the ten scans using Varian Eclipse^TM^ Treatment Planning System version 10.0.39, Varian Medical Systems, Palo Alto, USA. This was a contouring training exercise to ensure the guidelines would be applicable to patients with differing anatomy. Two cardiologists (KR and SM) reviewed, and made minor modifications, to the contours. The CT dataset most representative of typical anatomy, with minimal motion artefact, was selected as the atlas CT scan. Written guidelines for consistent contouring were jointly developed by two oncologists (FD, CT) and two cardiologists (KR, SM).

### Atlas description

#### The right and left atrioventricular grooves on the surface of the heart

**Fig. 1:**
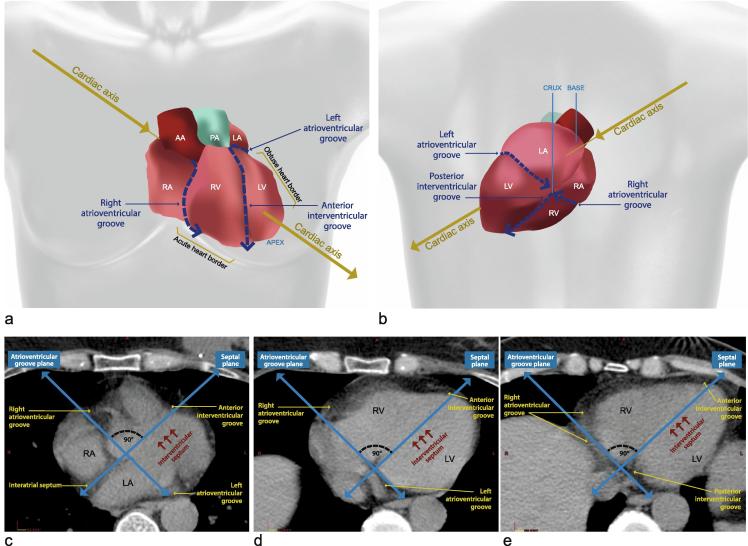
Identifying the atrioventricular and inter-ventricular grooves. (a and b) Anterior (a) and posterior (b) view of the whole heart illustrating the cardiac axis, the cardiac chambers, and the atrioventricular and inter-ventricular grooves. The cardiac axis projects through the centre of the base of the heart (the left atrium) towards the cardiac apex. The acute heart border is the horizontal heart border extending from the lower right edge of the heart to the apex, which is formed mainly by the right ventricle. The obtuse heart border separates the sterno-costal and left surfaces of the heart extending from the left atrium to the cardiac apex. (c–e) Axial CT images at the level of the inter-atrial septum (c), the proximal inter-ventricular septum (d) and the distal inter-ventricular septum (e). The atrial and ventricular septa define the septal plane. The plane defined by the atrioventricular grooves is usually perpendicular to the septal plane [11-12]. The septal plane may be marked with a line through the fat space between the right and left atria proximally, using the ruler tool on the treatment planning system. This represents the location of the interatrial septum which is usually clearly visible on CT (c). Scrolling down to the level of the ventricles this line overlies the interventricular septum and points just medial to the cardiac apex (d, e). Once the interventricular septum is located, the anterior and posterior interventricular grooves may be identified as they correspond to the anterior and posterior limits of the interventricular septum (d, e). The plane corresponding to the atrio-ventricular groove is approximately at right angles to the septal plane. The right and left atrio-ventricular grooves are usually identifiable since they are filled with fat (c–e). Abbreviations: PA: pulmonary artery, AA: ascending aorta, RA: right atrium, LA: left atrium, LV: left ventricle, RV: right ventricle.

Identification of the grooves that divide the heart into four chambers can help when contouring cardiac segments. The atrioventricular grooves form a continuous sulcus separating the atria from the ventricles (Fig. 1 a and b). The right atrioventricular groove begins anteriorly and superiorly, behind the ascending aorta. It then descends on the anterior surface of the heart and continues around the acute heart border (Fig.1a). The left atrioventricular groove originates with the right atrioventricular groove superiorly behind the aorta. The first part is obscured by the aorta and the pulmonary artery. It then curves around the obtuse border of the heart to connect with the other end of the right atrioventricular groove at the crux of the heart, which is the point on the posterior surface of the heart where the four chambers of the heart intersect forming a cross (Fig.1b).

#### The anterior and posterior interventricular grooves on the surface of the heart

The interventricular grooves are surface depressions on the myocardium which lie over the interventricular groove. The anterior interventricular groove descends on the anterior surface of the heart between the left and right ventricles to the apex. It is near, and almost parallel to, the obtuse heart border (Fig.1a). The posterior interventricular groove starts at the crux of the heart and runs along the inferior surface of the heart, which itself runs horizontally along the diaphragm, sloping down and forwards towards the apex (Fig.1b) [10], [11], [12].

#### The atrioventricular and interventricular grooves on axial CT scan

On CT it can be difficult to identify the interventricular septum, as the ventricles usually appear homogenous. It is useful to mark out two pre-defined planes: the septal plane, and the atrioventricular groove plane. These divide the heart approximately into the four chambers, and define the limits of the left ventricle (Fig.1c–e).

#### Left ventricular segmentation

**Fig. 2:**
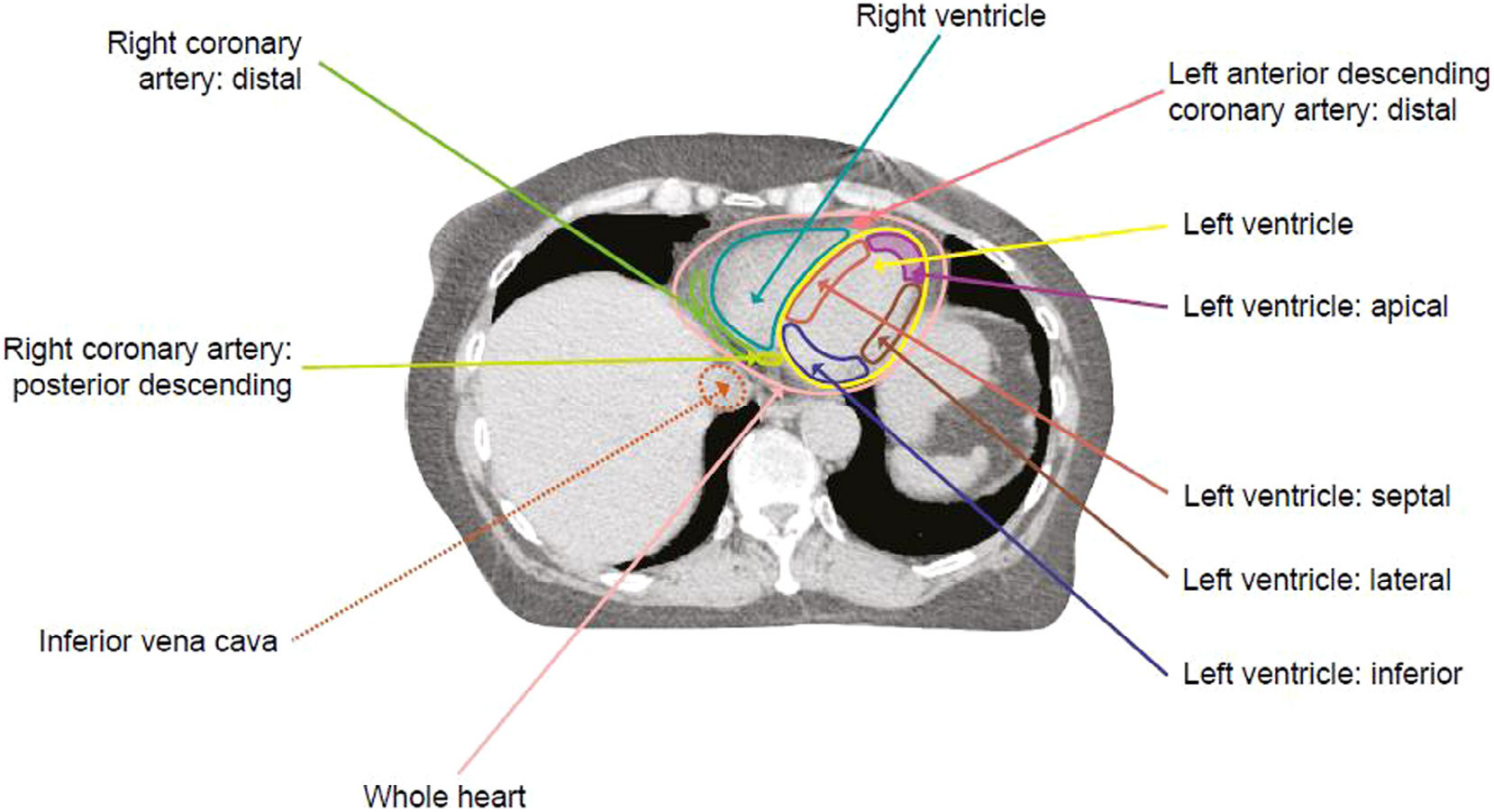
Axial radiotherapy CT planning image at the level of the left ventricle inferiorly showing contouring of the coronary arterial and left ventricular myocardial segments. See Supplementary Figs. 1–3 for further images.

The apical segment comprises the lowermost extent (the distal third) of the left ventricle. The attachment of the right ventricular wall to the left ventricle separates the septum from the apical, anterior and inferior free walls. The inferior segment extends from the mitral valve superiorly to join the apex inferiorly. The lateral and anterior segments form the lateral and superior walls of the ventricle respectively and extend inferiorly to meet the apex (Table 1, Fig. 2, Supplementary Figs. 1–3) [13], [14]. The average septal wall thickness in females is 1.1 cm for end-systole and 0.8 cm for end-diastole and the average posterior wall thickness is 1.4 cm for end-systole and 0.8 cm for end-diastole [15]. In the atlas, we set the thickness of the left ventricular wall as 1 cm throughout.

#### Coronary arterial segmentation

The left main coronary artery (LMCA) arises above the left aortic valve cusp and the right coronary artery (RCA) above the right cusp anteriorly. The levels at which these coronary arteries appear to originate may vary because patients may be scanned in different positions, or because the orientation of the cardiac axis may vary from patient to patient. Both these factors affect the orientation of the aorta relative to the axial CT slice. For nine of the ten CT scans in our study the RCA originated 4–6 axial CT slices (12–18 mm) inferior to the LMCA. For the remaining scan, the RCA originated on the same slice as the LMCA.

**Fig. 3:**
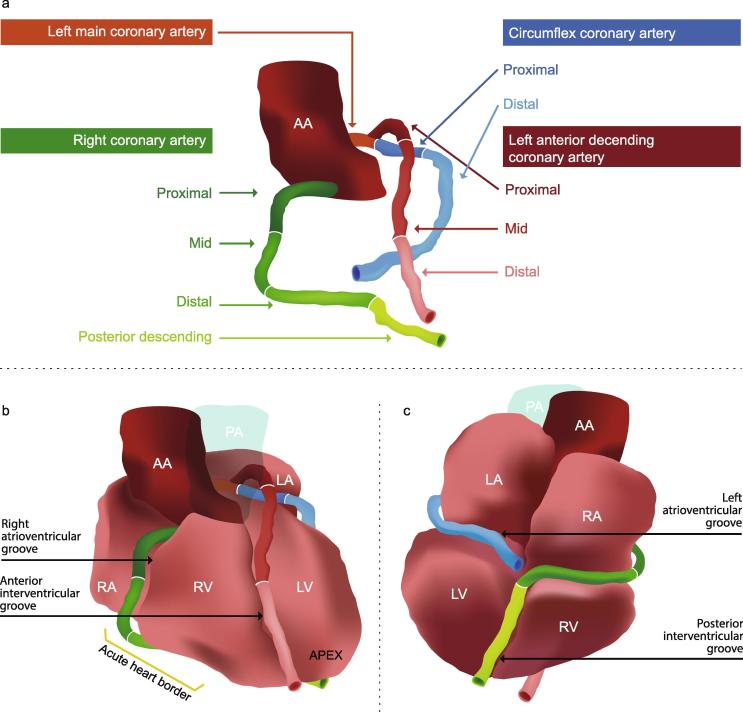
Coronary artery segmentation. 3D representation of the segments of the three main coronary arteries in relation to the atrial and ventricular chambers, ascending aorta and pulmonary artery. *Abbreviations:* PA: pulmonary artery, AA: ascending aorta, RA: right atrium, LA: left atrium, LV: left ventricle, RV: right ventricle.

The coronary arteries run in the atrioventricular and interventricular grooves. The LMCA passes between the pulmonary artery and left atrium and branches into the circumflex coronary artery (Cx) and the left anterior descending coronary artery (LADCA). The LADCA descends in the anterior inter-ventricular groove to the apex of the heart. The Cx descends around the left side of the heart in the left atrioventricular groove to the posterior surface as far as the crux. The RCA travels in the right atrioventricular groove and continues inferiorly around the acute heart border to the posterior surface towards the crux. The posterior descending artery (which usually originates from the RCA) runs from the crux of the heart in the posterior inter-ventricular groove towards the apex (Table 2, Fig. 2, Fig. 3, Supplementary Figs. 2–3) [16], [17], [18].

The diameter of each main coronary vessel decreases from proximal to distal. An angiography study of 90 women showed that the average coronary arterial luminal diameter was 3.2 mm [19]. In our atlas each coronary artery was contoured with 4 mm diameter throughout its entire length. The vessels were not tapered because of uncertainties in dose estimation for very small segments in CT planning.

### Atlas evaluation

Six oncologists specializing in breast or thoracic radiotherapy (DC, OM, RJ, SV, FB, and CT) tested the atlas. They received the written guidelines, atlas images and the atlas CT-scan in DICOM-format. They then contoured the left ventricle, the five left ventricular segments and the ten coronary arterial segments.

Contouring variability was assessed using 3D Slicer version 4.4 and the extension Slicer RT software to compute concordance metrics [20]. The atlas contours were selected as the “reference” contours, to which all the observer contours were compared. First, to assess contour overlap, the Sørensen-Dice similarity coefficient (DSC = 2*Z*/(*X* + *Y*)) was measured, where *X* is the reference atlas contour, *Y* is the observer contour, and *Z* is the region shared between the contours. A value of 1.0 indicates perfect concordance, and a value of 0 indicates no concordance. Second, to assess the separation between the contour surfaces, the directed Hausdorff average distance (${\overset{\to }{d}}_{H\text{,}\mathrm{avg}}$) was measured. This is the distance from each point (x) on the reference contour (X) to its closest point (y) of the observer contour (Y) averaged over all points on the reference contour: ${\overset{\to }{d}}_{H\text{,}\mathrm{avg}}(X\text{,}Y)=\frac{1}{\left|X\right|}\sum_{x\in X}\min_{y\in Y}d(x\text{,}y)$, where d is the distance between *x* and y and |*X*| is the number of points on *X* [21]. The mean DSC and the mean ${\overset{\to }{d}}_{H\text{,}\mathrm{avg}}$ refer to the arithmetic mean of the measurements recorded for each of the six observer contour/reference contour comparisons.

To assess the dosimetric consequences of variation in contouring, six breast cancer radiotherapy regimens were reconstructed. These delivered 95% of the prescribed dose (50 Gy in 2 Gy fractions) to the breast PTV and 85% to the internal mammary and medial supraclavicular fossa nodes. Left and right-sided regimens were reconstructed for: (1) conformal partially wide tangential technique (PWT), (2) conformal oblique parasternal photon technique (OPP) and (3) conventional mixed electron/photon direct parasternal technique (E^_^/MV) [22]. The analytical anisotropic algorithm was used for dose calculations using the 0.1 cc calculation volume grid. For each of the six regimens, the mean dose to each cardiac segment was recorded.

## Results

Contour overlap was greater for the left ventricular segments than for the coronary arterial segments (Table 3, Supplementary Fig. 4). The mean DICE coefficient for the left ventricle was 0.91 and ranged between 0.60 and 0.73 for the left ventricular segments, indicating considerable overlap between the contours. The mean ${\overset{\to }{d}}_{H\text{,}\mathrm{avg}}$ ranged from 1.5 to 2.2 mm. For the coronary arterial segments, the mean DICE coefficient was between 0.10 and 0.53. Despite poor overlap, the separation between the contours was not substantial as the *d_H_,_avg_* was only a few mm, ranging from 1.3 to 5.1 mm.

Spatial variation in contouring resulted in differences to the doses received by each segment from the six radiotherapy regimens reconstructed. There were 96 regimen/segment combinations: 48 for left-sided regimens (Table 3) and 48 for right-sided regimens (Supplementary Table 1). For 67 of the 96 regimen/segment combinations, segments were not near the fields. Where this occurred, doses to most segments were <3 Gy, and the differences between the minimum and maximum doses for the observers were usually <1 Gy. For six regimen/segment combinations, the segment was in the high dose area, and not near any field borders (Table 3 italics). This occurred only for one regimen, left E^_^/MV radiotherapy, where the LV inferior, LV septal, LMCA, LADCA proximal, LADCA mid and the Cx proximal were in the radiation field for all observers. Segment doses were between 17.3 and 28.1 Gy for all observers, and the difference between the minimum and maximum doses was always less than 2 Gy. For the other 23 regimen/segment combinations, segments were on or near field borders (Table 3, Supplementary Table 1 bold type), resulting in differences of 2.2 to 21.8 Gy between the minimum and maximum estimated doses, with standard deviations ranging from 0.4 to 8.8 Gy.

## Discussion

In this study we present an atlas for contouring segments of the left ventricle and the coronary arterial tree on radiotherapy planning CT scans. This will facilitate consistent reporting of cardiac doses in radiotherapy research studies and in clinical trials so that radiation doses to these parts of the heart may be related to clinical outcome data. Such dose–response relationships may help clinicians assess the risks of damage to particular cardiac segments.

Spatial overlap averaged over the six observers was more than 60% for left ventricular segments but less than 50% for most coronary artery segments and the separation between the contours averaged over the six observers was larger for coronary artery segments (1.3–5.1 mm) than for left ventricle segments (1.5–2.2 mm). Poor overlap of the coronary arterial segment contours was expected because they are (1) difficult to outline reproducibly being long, narrow, irregularly-shaped structures and (2) small in volume. As small contours cover a smaller area for a given CT area they are less likely to overlap. Assessing the effects of differences in contours on radiotherapy dose we found that dose variation was much greater for segments near a field edge than for other segments. Our findings are similar to those of Lorenzen [23] and Feng [10] who also reported inter-observer variability in contouring was much larger for cardiac structures that were near field edges than for other structures that were further away. Similar cardiac segment dose variation is likely to occur in modern regimens which have steep dose gradients, such as proton therapy. For hypofractionated regimens, total target dose is lower so variation in absolute cardiac segment doses will be lower. Nevertheless, variation in EQD2 would be similar.

Our atlas aims to minimize dose variation from one source of uncertainty: differences in contouring. Other sources of uncertainty also need to be considered when estimating cardiac doses. Errors in set-up, dose-calculation algorithms, and intra-fraction motion secondary to breathing and the cardiac cycle all contribute to dose uncertainty, even where individual CT scans are available. Where individual CT scans are not available and a representative patient is used for retrospective dose estimation, a further source of error is variation in patient anatomy.

Our study has a number of strengths. First, the atlas was developed jointly between oncologists and cardiologists who agreed on the segments and instructions for contouring them. Second, six observers from three different countries contoured all the cardiac segments so a range of oncologists were represented. Third, the relevance of the segments to subsequent injury was confirmed by echocardiograms and angiograms from the cardiology records of 500 women. A number of models exist for segmenting the left ventricle. One such model includes 17 segments [14] which are subvolumes of the five main segments described in our atlas. Contouring 17 left ventricular segments reproducibly would be challenging and estimating doses to these subvolumes would only be useful after confirming that the cardiac outcome data, to which the doses will be related, describes those particular subvolumes. Fourth, coronary artery segment doses give information on the spatial location of the high dose regions within the artery. In the future, oncologists may wish to avoid certain segments if they are shown to be more sensitive to radiation-related damage than others, or if there is evidence that injury to certain segments is associated with worse outcomes e.g. LADCA proximal stenosis usually has a worse outcome than LADCA mid or distal stenosis [24]. Finally this atlas is based on a non-contrast CT planning scan and so it is useful for contouring both on non-contrast scans, which are routinely acquired for patients with breast cancer, and contrast-enhanced scans, which are routinely acquired for patients with Hodgkin lymphoma, lung and oesophageal cancer. Contrast may improve the accuracy of coronary artery identification, and the same definitions may be followed to identify segment limits. One study has shown that contrast helps to localize the main coronary arteries, but only for the proximal one third of the coronary arteries [25] and in the University of Michigan atlas, contrast made no difference to inter-observer variation in dose reporting [10].

Our study has some limitations. First, it was not possible to compare observer contours before and after access to the atlas. Oncologists are not trained in cardiac contouring so without the atlas the observers would not have been able to contour most segments. Second, the atlas is based on one CT-planning scan but anatomy differs between patients. For example for some women the LADCA proximal has a loop configuration [39] so the atlas would not correctly identify the LADCA proximal position for them. Also, the location of the interventricular septum may vary. For example in the few patients with dilated cardiomyopathy, the interventricular septum may not be in line with the interatrial septum, so may not be correctly identified using the instructions in Fig. 1.

This atlas provides definitions for contouring the coronary arterial and left ventricular segments on non-contrast CT-planning scans. There may be ways to improve identification of these segments in the future. First, contrast improves the ability to visualize the main coronary arteries but there are limitations as described above. Second, the use of respiratory and cardiac-gated images reduces blurring due to respiratory motion and the dynamic cardiac cycle but this approach may not be practical if acquiring CT-planning scans on a large number of patients. Third, left ventricular segments may be easier to locate by fusing cardiac magnetic resonance scans to CT-planning scans but, as yet, there is no validated algorithm for this. Fourth, it may be possible to develop a tool for automatically delineating these structures in the future. Lastly, once radiotherapy commences, additional information from daily image guided radiotherapy may further improve the accuracy of the cardiac segment doses.

This study provides an atlas enabling contouring of left ventricular and coronary arterial segments on radiotherapy CT-planning scans which may be applied to radiotherapy that involves exposure of the heart. The use of consistent guidelines for segment contouring will facilitate future research studies investigating relationships between segment doses and cardiac outcomes.

## Conflict of interest statement

None.

## Figures and Tables

**Table 1:** Left ventricular myocardial segmentation: use of the traditional 17-segment model to derive five segments for the cardiac contouring atlas.

Present study	17-Segment Model [13], [14]	
Segment name	Segment number*	Segment name
Anterior	1	Basal anterior
	7	Mid anterior

Lateral	5	Basal inferolateral
	6	Basal anterolateral
	11	Mid inferolateral
	12	Mid anterolateral

Apical	13	Apical anterior
	14	Apical septal
	15	Apical inferior
	16	Apical lateral
	17	Apex

Septal	2	Basal anteroseptal
	3	Basal inferoseptal
	8	Mid anteroseptal
	9	Mid inferoseptal

Inferior	4	Basal inferior
	10	Mid inferior

⁎ The traditional 17-segment model divides the left ventricle into basal, mid and apical thirds. Segment numbers relate to the position of the segments, extending from the base to the apex.

**Table 2:** Definition of the ten atlas coronary artery segments.

Atlas coronary artery segments	Contour definition [16], [17]
Left main coronary artery	From the left lateral ascending aorta running between the left atrium and the pulmonary artery to the bifurcation into left anterior descending and circumflex coronary arteries (∼1.5 cm in length)

Left anterior descending coronary artery	
Proximal	The proximal 1/5th of the vessel, from the end of the left main coronary artery passing anteriorly behind the pulmonary artery
Mid	The mid 2/5th of the vessel descending anterolaterally in the anterior interventricular groove
Distal	The distal 2/5th of the vessel running in the interventricular groove and extending to the apex

Circumflex coronary artery	
Proximal	From the end of the left main coronary artery running in the left atrioventricular groove for 2 cm in length
Distal	From the end of the circumflex proximal running along the left atrioventricular groove posteriorly extending to the crux of the heart

Right coronary artery	
Proximal	From the anterior aspect of the ascending aorta descending in the right atrioventricular groove to one-half the distance to the acute heart border*
Mid	From the end of right coronary artery proximal to the acute heart border*
Distal	From the acute heart border* running medially along the right atrioventricular groove posteriorly to the crux of the heart
Posterior descending	From the crux of the heart to the apex running in the posterior interventricular groove to the tip of the apex

* The acute heart border is the horizontal heart border extending from the lower right edge of the heart to the apex formed mainly by the right ventricle.

**Table 3:** Variation in spatial measurements and doses from left breast cancer regimens for cardiac segments contoured by six observers on one CT-planning dataset.

Cardiac segments	Spatial variation			Dose variation (Gy), left-sided regimens		
	Mean (SD), range of values			Mean (SD), range of values		
	DICE coefficient	Hausdorff average distance (mm)	Structure volume (cc)	PWT	OPP	E^_^/MV
Left ventricle	0.91, 0.89–0.94	1.3, 0.9–1.8	170.4, 149.8–181.6	6.7 (0.2), 6.4–7.0	8.2 (0.2), 7.9–8.6	15.6 (0.3), 15.0–15.9
LV: apical	0.69, 0.65–0.78	1.5, 1.0–1.8	19.8, 13.1–25.1	**22.3 (2.8), 18.8**–**26.4**	**21.0 (2.1), 18.6**–**24.1**	**9.9 (0.8), 9.1**–**10.9**
LV: lateral	0.73, 0.67–0.77	1.6, 1.2–2.5	22.1, 18.1–25.4	**3.4 (1.1), 2.0**–**4.6**	**6.3 (1.6), 4.3**–**7.9**	**8.5 (1.1), 7.2**–**9.7**
LV: inferior	0.65, 0.50–0.74	2.1, 1.6–2.7	13.0, 9.1–16.4	0.8 (0.1), 0.7–0.8	1.0 (0.0), 1.0–1.1	*17.6 (0.2), 17.3*–*17.9*
LV: septal	0.60, 0.45–0.76	2.2, 1.4–2.9	21.0, 15.6–29.4	**4.5 (0.8), 3.1**–**5.3**	**5.5 (1.7), 3.7**–**6.9**	*22.0 (0.3), 21.6*–*22.5*
LV: anterior	0.65, 0.60–0.78	1.6, 1.7–2.3	12.8, 7.3–23.0	**9.6 (4.1), 5.1**–**17.3**	**14.0 (3.9), 9.3**–**20.7**	**17.4 (1.3), 15.2**–**18.9**

LMCA	0.45, 0.09–0.76	1.4, 0.5–2.4	0.4, 0.2–0.6	1.5 (0.1), 1.1–1.2	1.3 (0.1), 1.1–1.3	*19.1 (0.4), 18.5*–*19.5*
LADCA proximal	0.53, 0.34–0.72	1.3, 0.5–2.2	1.1, 0.7–2.0	2.5 (0.5), 1.9–3.2	3.1 (0.6), 2.3–4.0	*22.2 (0.6), 21.3*–*23.0*
LADCA mid	0.39, 0.23–0.53	1.5, 0.7–2.1	0.8, 0.4–1.0	**25.1 (2.4), 22.1**–**28.5**	**27.0 (2.1), 23.7**–**29.1**	*28.1 (0.5), 27.4*–*28.9*
LADCA distal	0.23, 0.03–0.39	2.4, 1.2–4.6	0.6, 0.2–0.9	**35.8 (2.8), 33.5**–**40.0**	**32.0 (3.4), 28.0**–**37.8**	**21.8, (8.8), 12.1**–**33.9**

Cx proximal	0.25, 0.04–0.65	2.7, 0.7–4.5	0.4, 0.2–0.5	1.3 (0.1), 1.2–1.5	1.5 (0.1), 1.4–1.7	*19.8 (0.3), 19.5*–*20.3*
Cx distal	0.18, 0.06–0.31	3.4, 2.1–5.5	2.1, 0.7–4.5	0.9 (0.0), 0.9–1.0	1.1 (0.1), 1.1–1.3	**17.4 (0.4), 16.9**–**18.0**

RCA proximal	0.35, 0.00–0.46	3.1, 2.2–6.2	0.5, 0.2–0.9	1.7 (0.1), 1.5–1.7	1.4 (0.1), 1.3–1.4	3.0 (0.5), 2.1–3.4
RCA mid	0.22, 0.00–0.31	3.7, 1.8–10.7	0.3, 0.2–0.6	1.0 (0.1), 0.9–1.1	0.7 (0.0), 0.7–0.8	1.4 (0.1), 1.3–1.4
RCA distal	0.44, 0.30–0.55	1.9, 1.1–3.8	1.2, 1.1–1.5	0.6 (0.1), 0.5–0.7	0.5 (0.0), 0.5–0.5	1.6 (0.2), 1.3–1.9
RCA post desc	0.10, 0.00–0.26	5.1, 3.0–7.6	0.9, 0.5–1.4	0.8 (0.2), 0.6–1.0	0.9 (0.1), 0.7–1.0	**14.9 (2.0), 11.8**–**16.8**

Dose variation results are highlighted in bold for structures located near a high dose-gradient and in italics for structures located within the high-dose areas.

*Definitions:*

*DICE coefficient*: 2*Z*/(*X* + *Y*), where *X* is the reference atlas contour, *Y* is the observer contour, and *Z* is the region shared between the contours. The mean DICE coefficient refers to the arithmetic mean of the measurements recorded for each of the six observer contour/reference contour comparisons.

*Hausdorff average distance*: the distance from each point (*x*) on the reference contour (*X*) to its closest point (*y*) of the observer contour (*Y*) averaged over all points on the reference contour: 1/|*X*| ∑*x*∈*X*min*y*∈*Y d*(*x*,*y*), where *d* is the distance between *x* and *y* and |*X*| is the number of points on *X*. The mean Hausdorff average distance refers to the average of the measurements recorded for each of the six observer contour/reference contour comparisons.

*Abbreviations:* PWT: partially wide tangential technique; OPP: oblique parasternal photon technique; E–/MV: mixed electron/photon direct parasternal technique; LV: left ventricle, LMCA: left main coronary artery, LADCA: left anterior descending coronary artery, Cx: circumflex coronary artery; RCA: right coronary artery; post desc: posterior descending.
