# Supplementary material for: A cardiac contouring atlas for radiotherapy
Source: Radiother Oncol. 2017 Mar;122(3):416–22. doi: 10.1016/j.radonc.2017.01.008 (PMC5356506; doi:10.1016/j.radonc.2017.01.008)
Supplement: Supplementary data — Supplementary Figs. 1–4, Table 1. [file mmc1.pdf]

Appendix A. Supplementary data

A Cardiac Contouring Atlas for Radiotherapy

Supplementary Fig. 1. Diagram of 17-segment myocardial segmentation model 2

Supplementary Fig. 2. Axial radiotherapy CT planning images showing contouring of the coronary arterial and left ventricular myocardial segments (slice thickness 3mm). 3

Supplementary Fig. 3. Magnified axial radiotherapy CT planning images showing contouring of the coronary arterial and left ventricular myocardial segments (slice thickness 3mm). 5

Supplementary Fig. 4. Cardiac substructure contours by six observers on one CT-planning scan at the level of the left ventricle superiorly (a) and inferiorly (b). 38

Supplementary Table 1. Variation in doses from right breast cancer regimens for cardiac segments contoured by six observers on one CT-planning dataset. 39

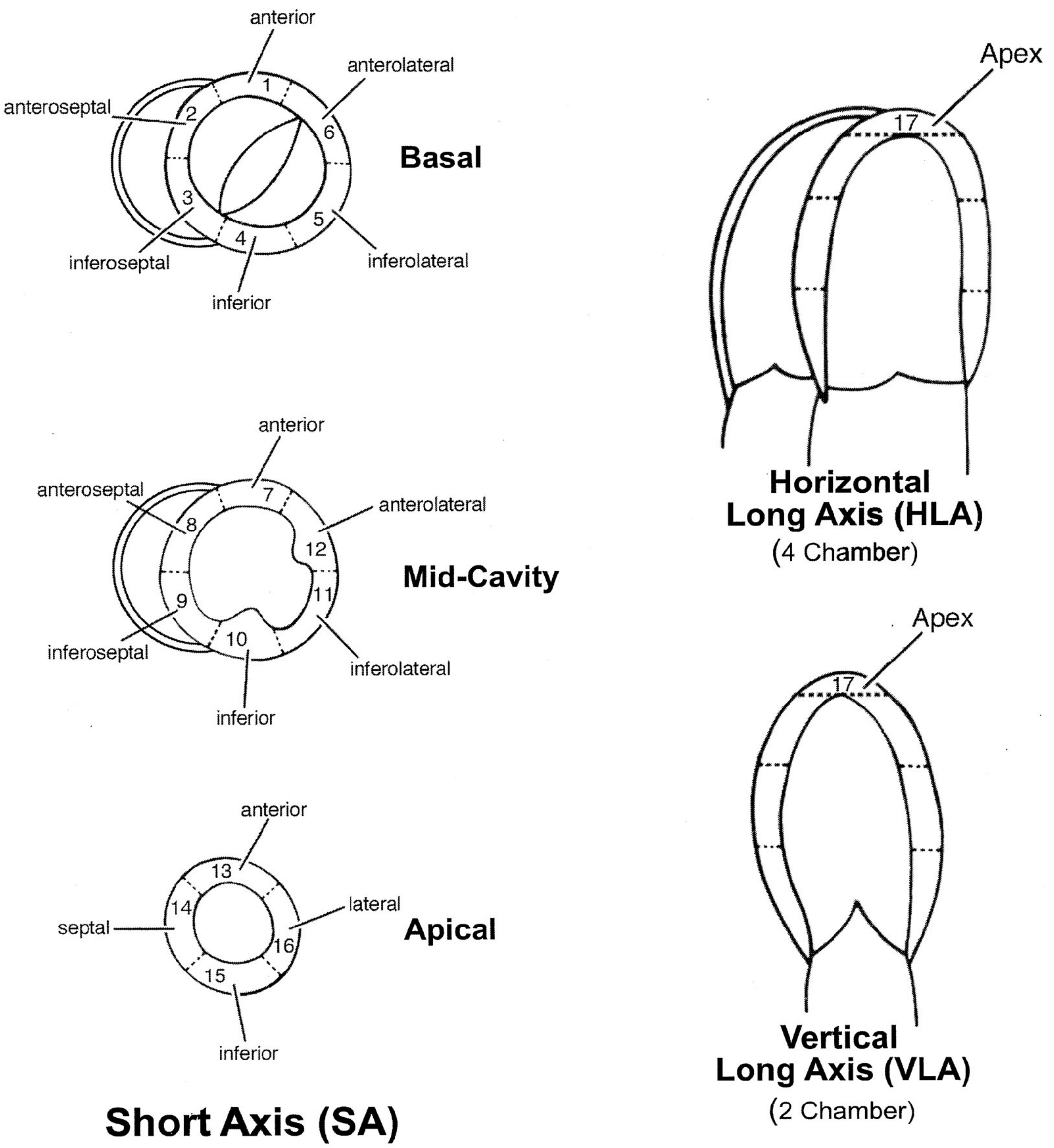

Supplementary Fig. 1. Diagram of 17-segment myocardial segmentation model.

Diagram of vertical long-axis (VLA, approximating the 2-chamber view), horizontal long-axis (HLA, approximating the 4-chamber view), and short-axis (SA) planes showing the name, location, and anatomic landmarks for selection of the basal (tips of the mitral valve leaflets), mid-cavity (papillary muscles), and apical (beyond papillary muscles but before cavity ends) short-axis slices for the recommended 17-segment system. All imaging modalities should use these same landmarks, when available, for slice detection.

Circulation : journal of the American Heart Association by American Heart Association Reproduced with permission of American Heart Association in the format Journal/magazine via Copyright Clearance Center. Order Detail ID: 70109125

http://circ.ahajournals.org/content/105/4/539

Supplementary Fig. 2. Axial radiotherapy CT planning images showing contouring of the coronary arterial and left ventricular myocardial segments (slice thickness 3mm).

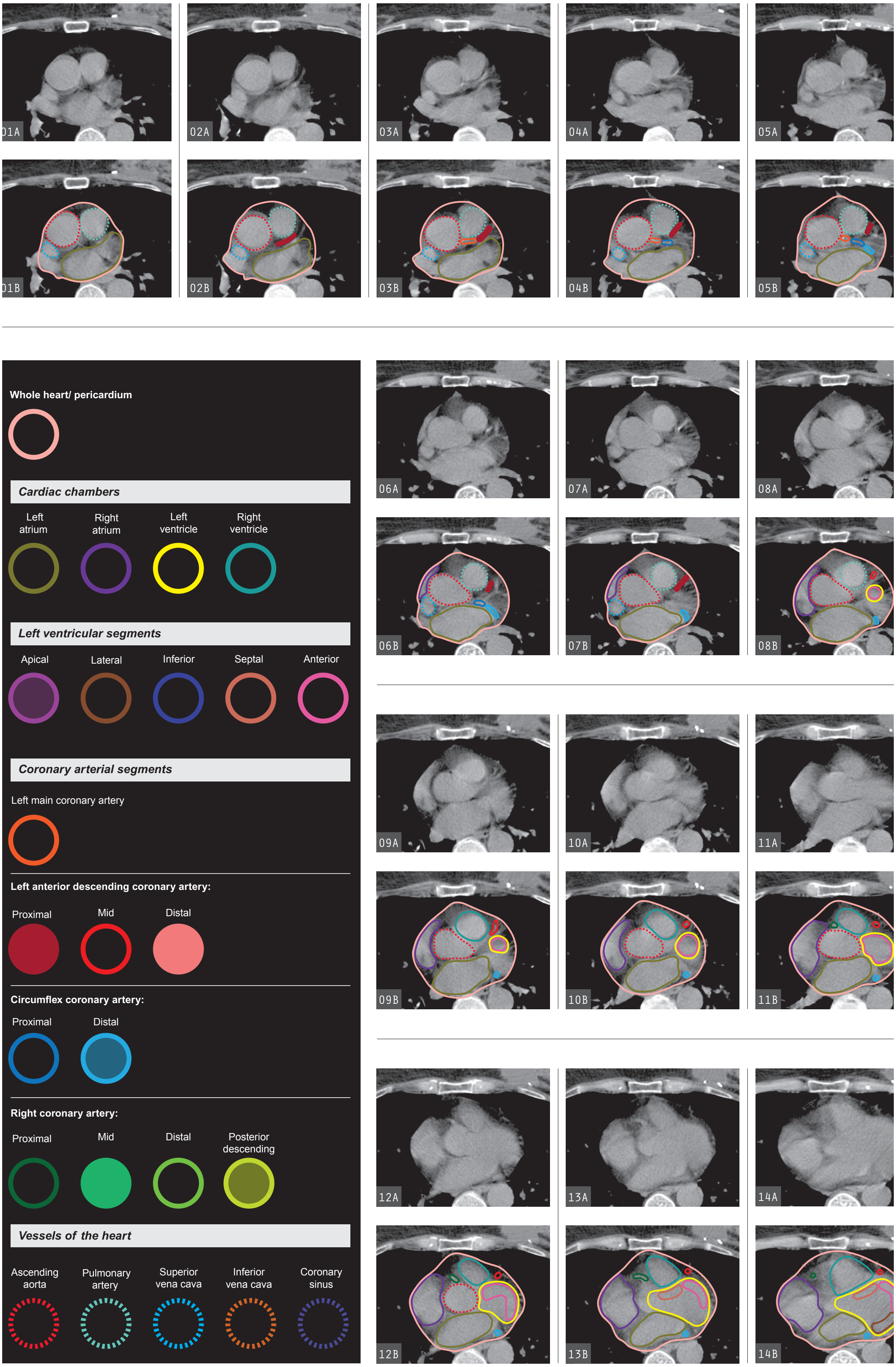

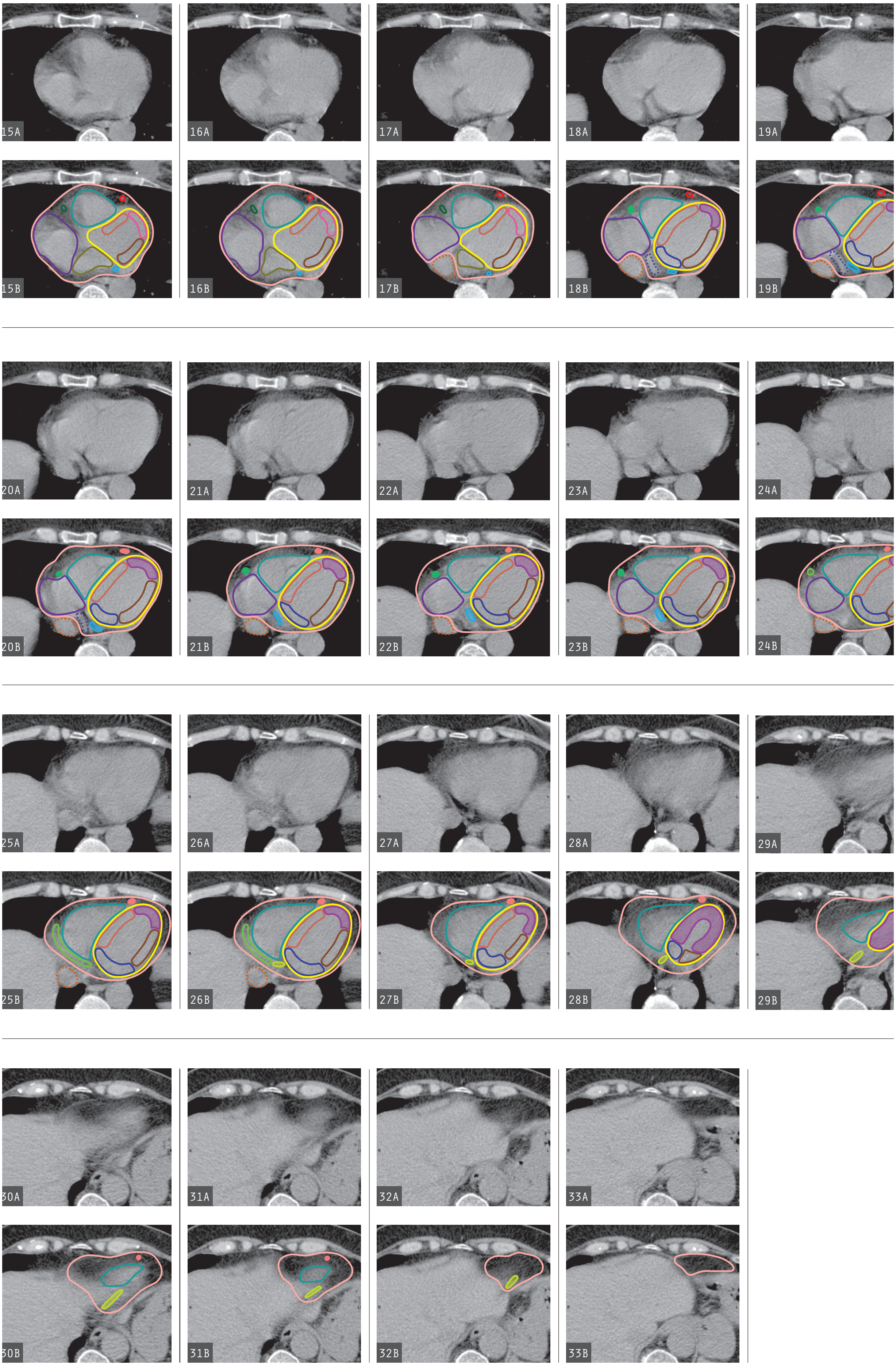
15A
16A
17A
18A
19A
15B
16B
17B
18B
19B
20A
21A
22A
23A
24A
20B
21B
22B
23B
24B
25A
26A
27A
28A
29A
25B
26B
27B
28B
29B
30A
31A
32A
33A
30B
31B
32B
33B

Supplementary Fig. 3. Magnified axial radiotherapy CT planning images showing contouring of the coronary arterial and left ventricular myocardial segments (slice thickness 3mm).

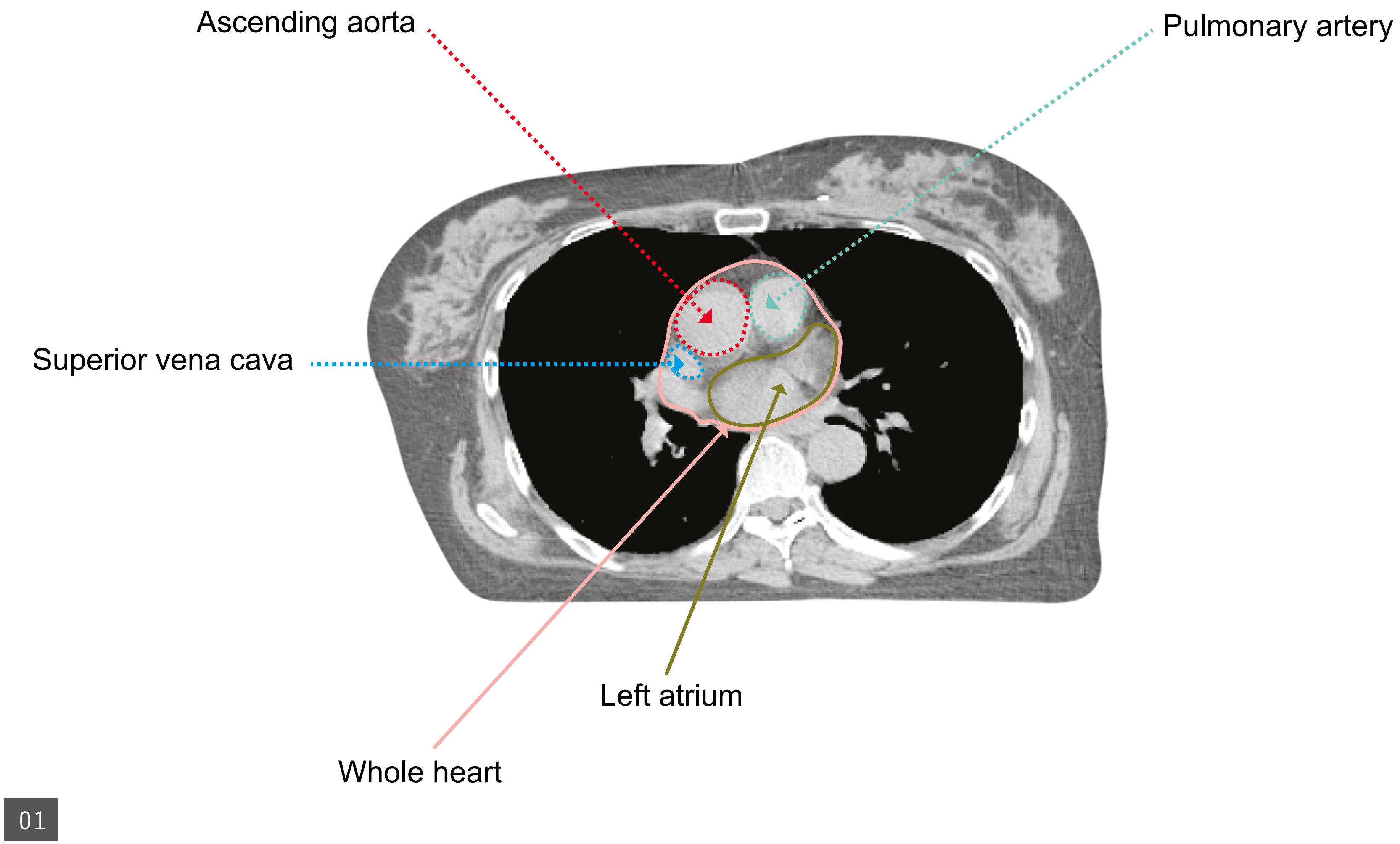

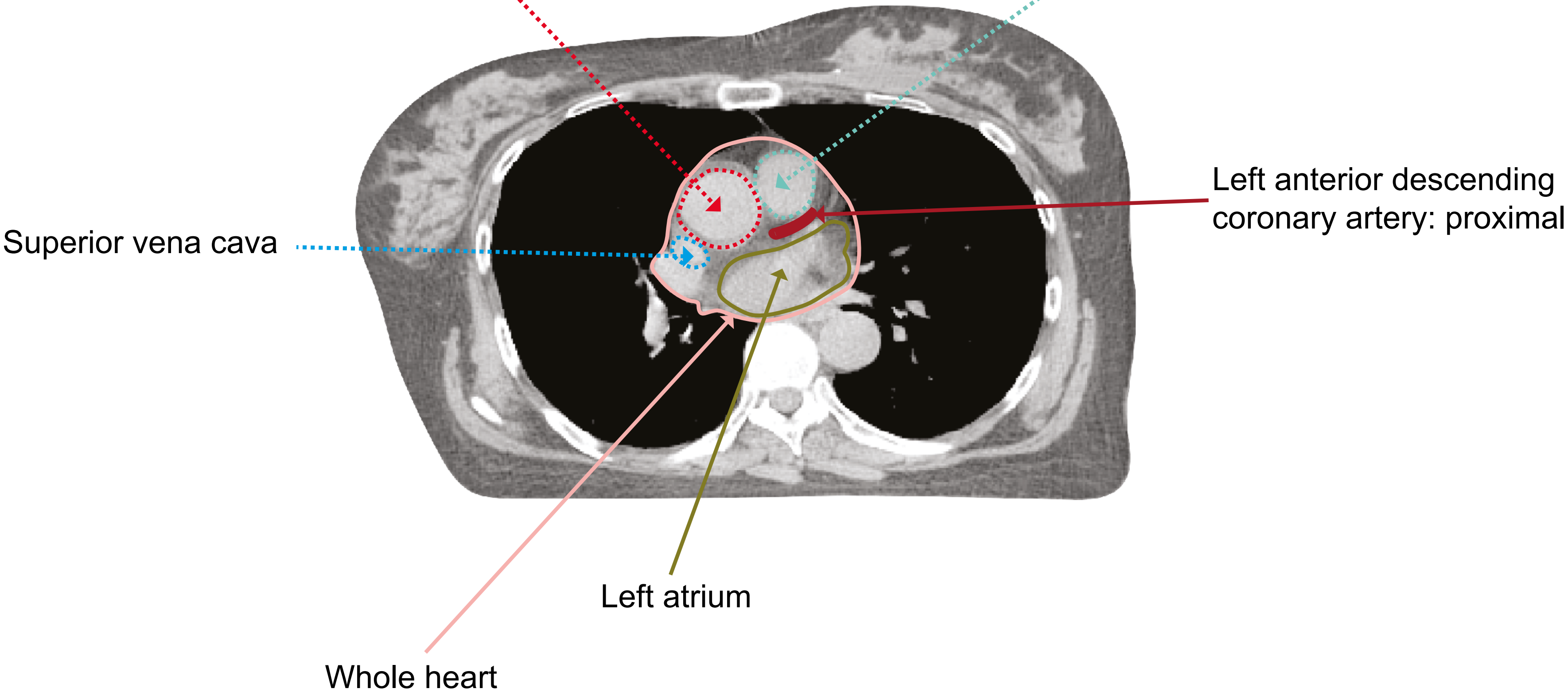
Ascending aorta
Pulmonary artery
Left anterior descending
coronary artery: proximal
Superior vena cava
Left atrium
Whole heart

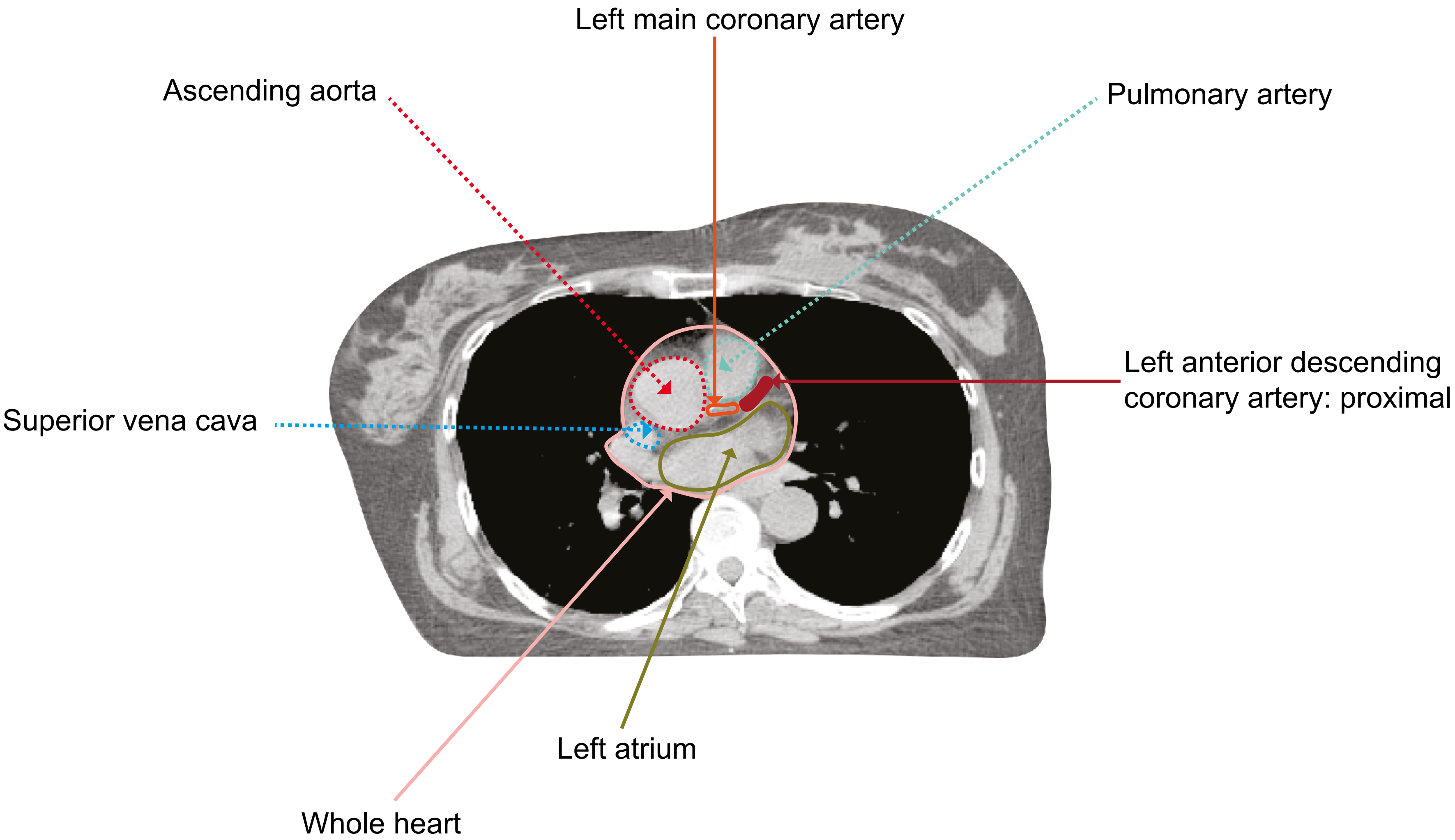
Left main coronary artery
Ascending aorta
Pulmonary artery
Left anterior descending coronary artery: proximal
Superior vena cava
Left atrium
Whole heart

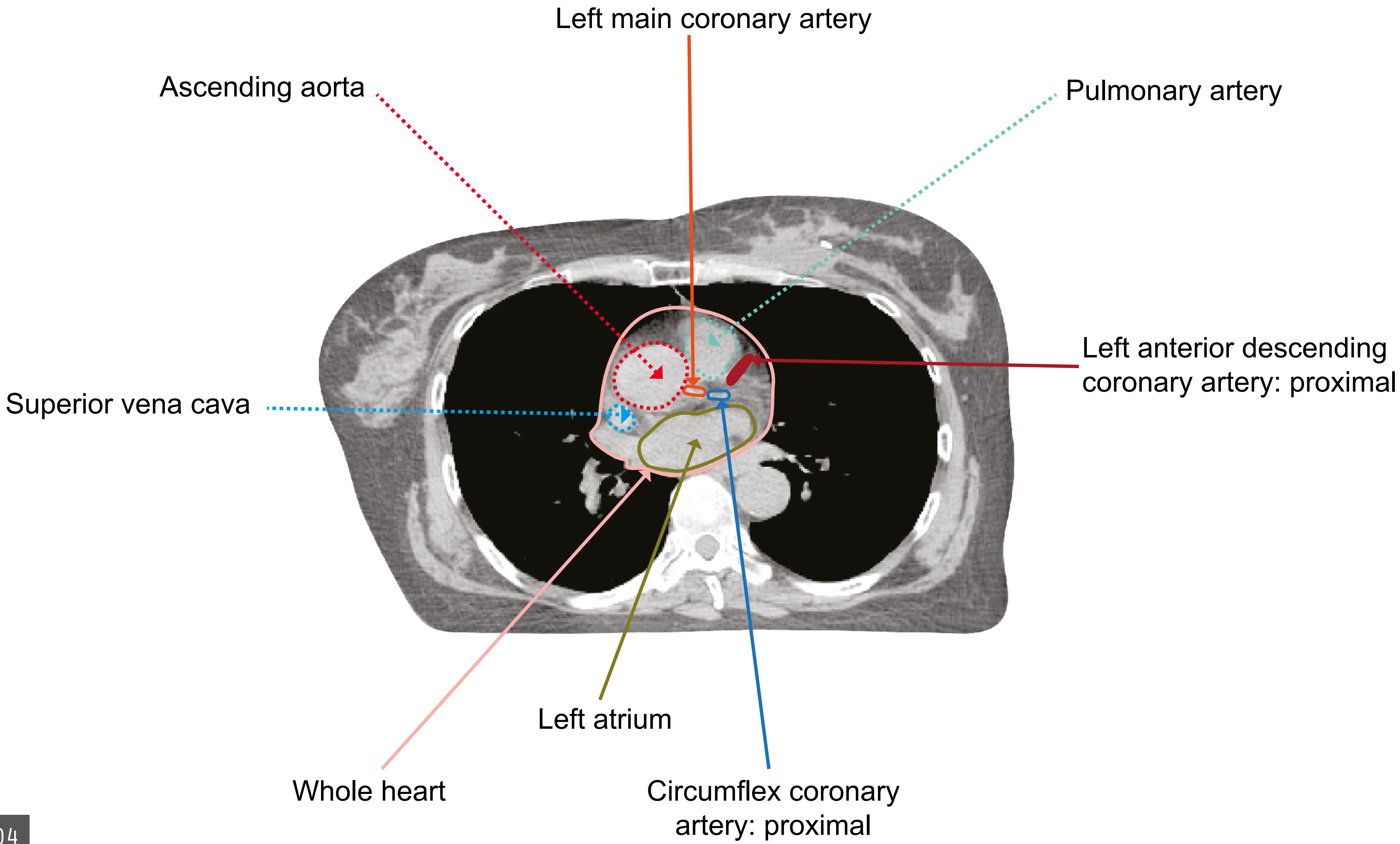
Left main coronary artery
Ascending aorta
Pulmonary artery
Left anterior descending coronary artery: proximal
Superior vena cava
Left atrium
Whole heart
Circumflex coronary artery: proximal

04

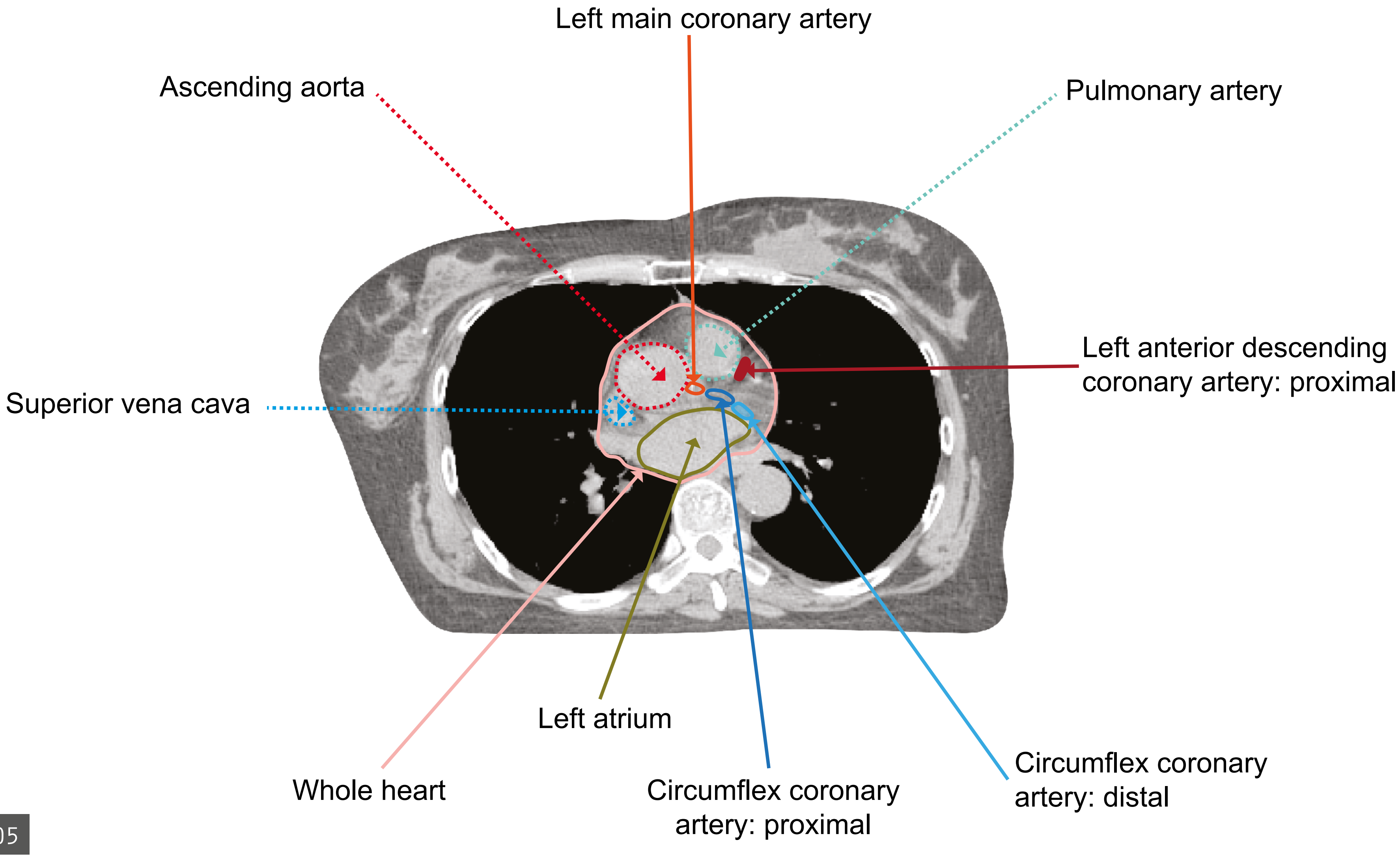
Left main coronary artery
Ascending aorta
Pulmonary artery
Left anterior descending coronary artery: proximal
Superior vena cava
Left atrium
Whole heart
Circumflex coronary artery: proximal
Circumflex coronary artery: distal

05

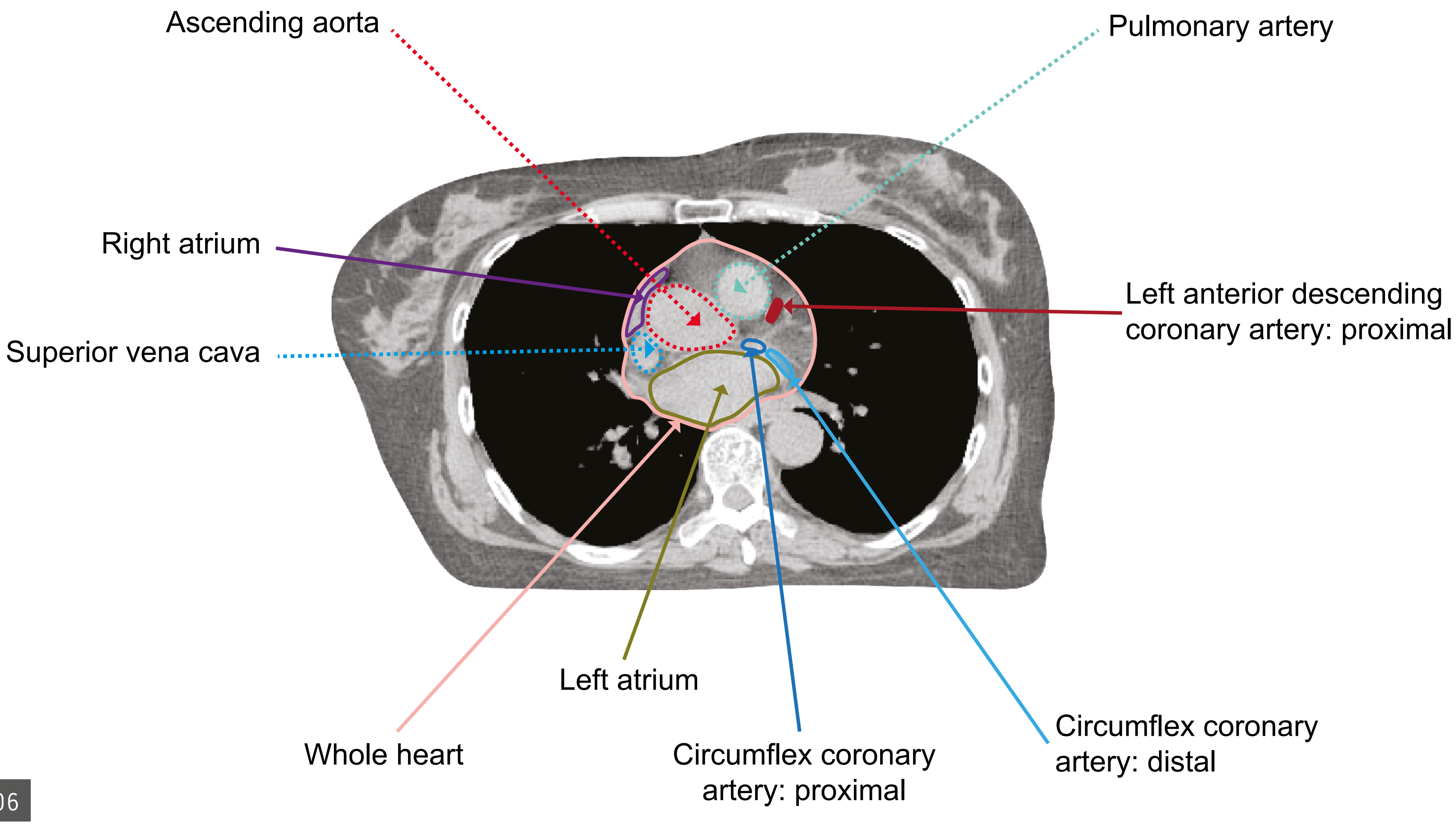
Ascending aorta
Pulmonary artery
Right atrium
Left anterior descending coronary artery: proximal
Superior vena cava
Left atrium
Whole heart
Circumflex coronary artery: proximal
Circumflex coronary artery: distal

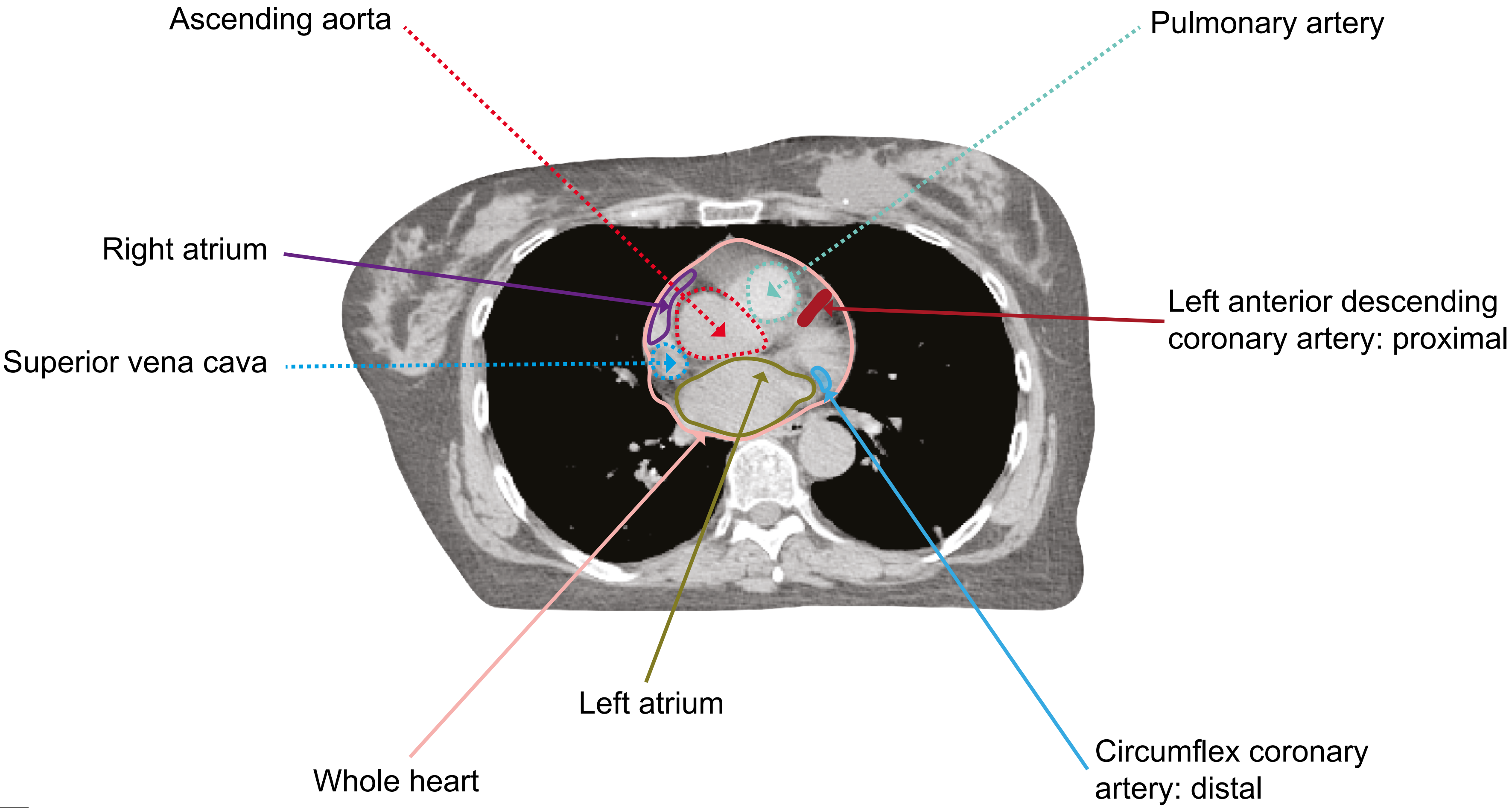
Ascending aorta
Pulmonary artery
Right atrium
Left anterior descending coronary artery: proximal
Superior vena cava
Left atrium
Whole heart
Circumflex coronary artery: distal

07

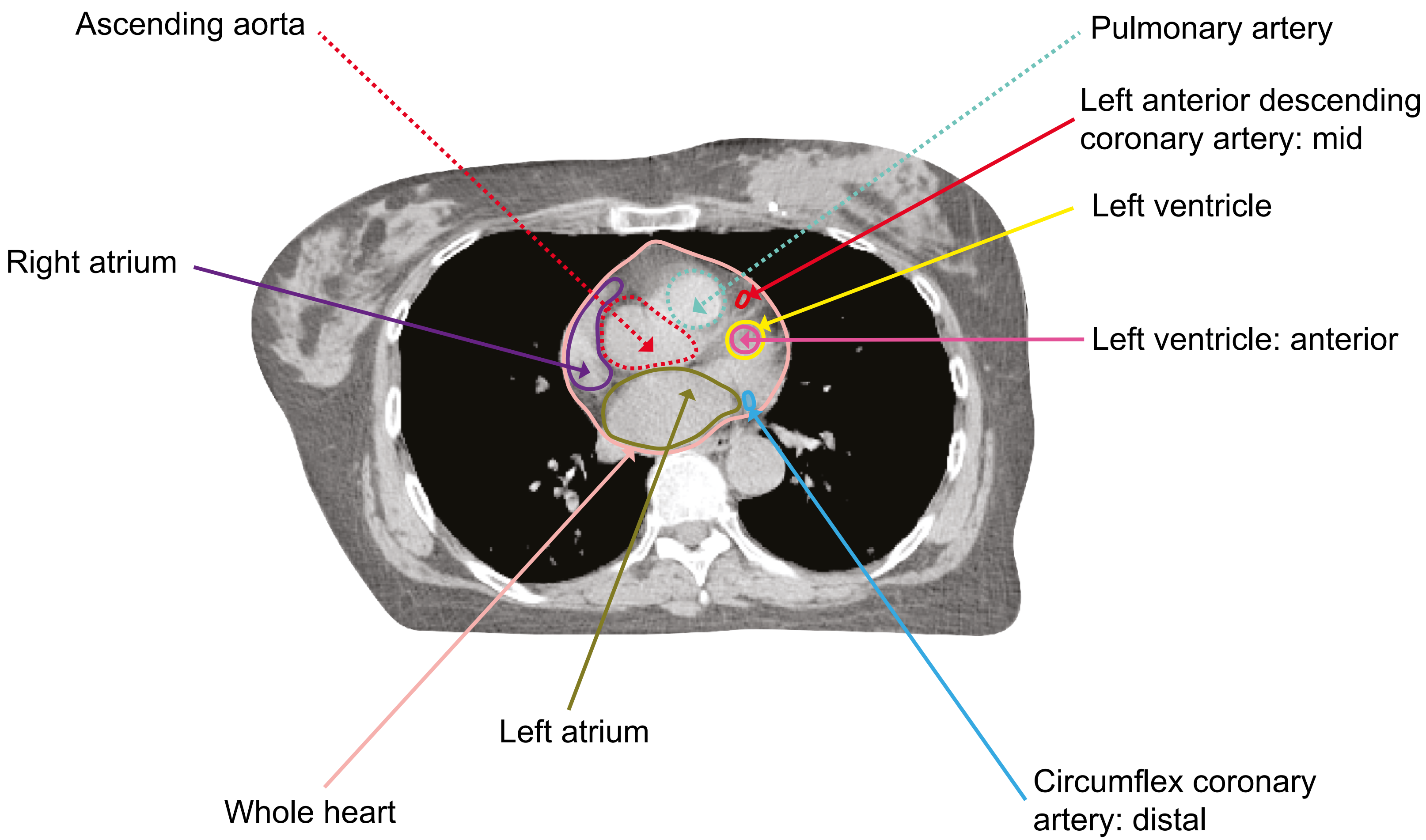
Ascending aorta
Pulmonary artery
Left anterior descending coronary artery: mid
Left ventricle
Right atrium
Left ventricle: anterior
Left atrium
Whole heart
Circumflex coronary artery: distal

08

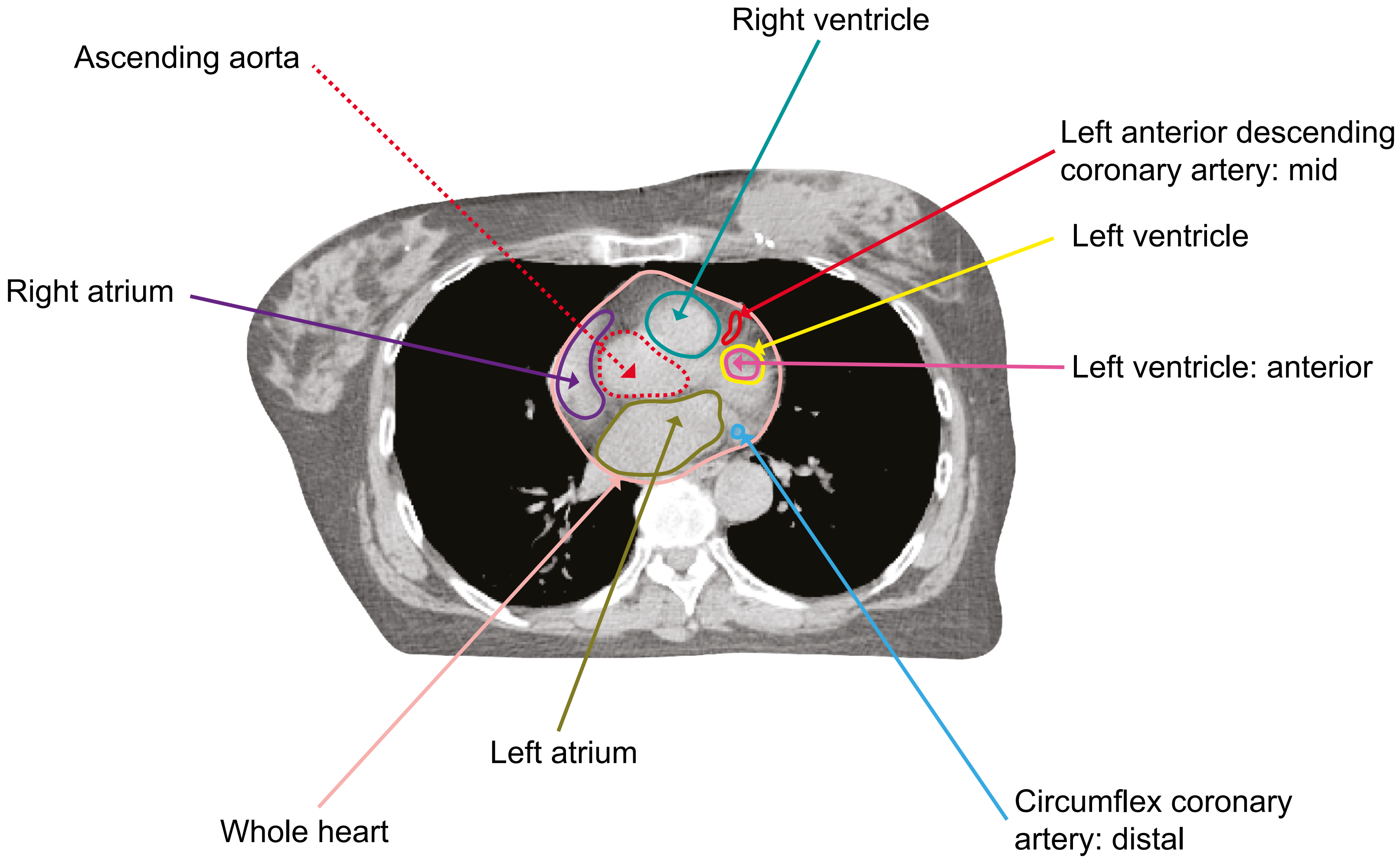
Right ventricle
Ascending aorta
Left anterior descending coronary artery: mid
Left ventricle
Right atrium
Left ventricle: anterior
Left atrium
Whole heart
Circumflex coronary artery: distal

09

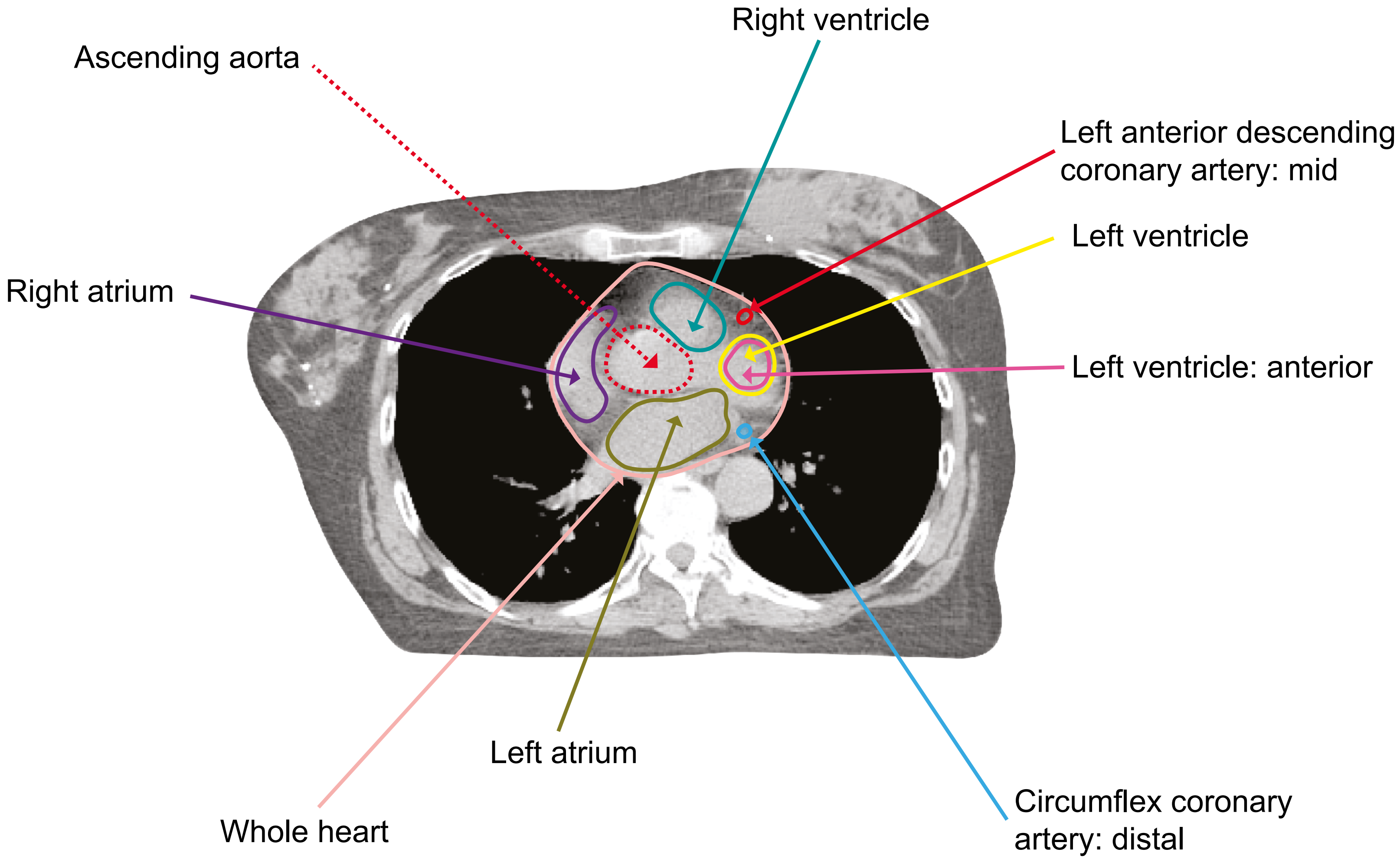

Ascending aorta
Right ventricle
Left anterior descending coronary artery: mid
Left ventricle
Right atrium
Left ventricle: anterior
Left atrium
Whole heart
Circumflex coronary artery: distal

10

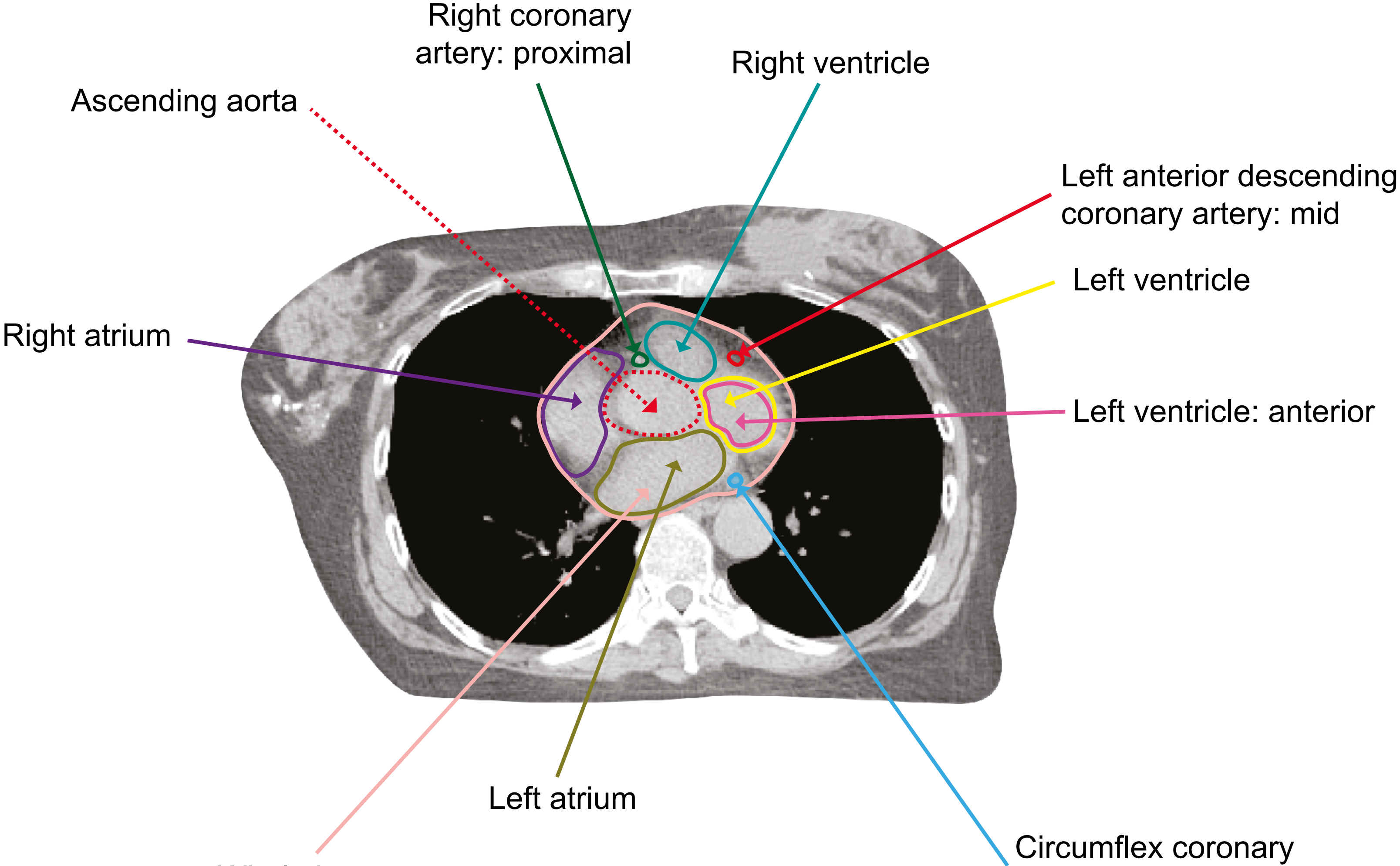
Right coronary artery: proximal
Right ventricle
Ascending aorta
Left anterior descending coronary artery: mid
Left ventricle
Right atrium
Left ventricle: anterior
Left atrium
Whole heart
Circumflex coronary artery: distal

11

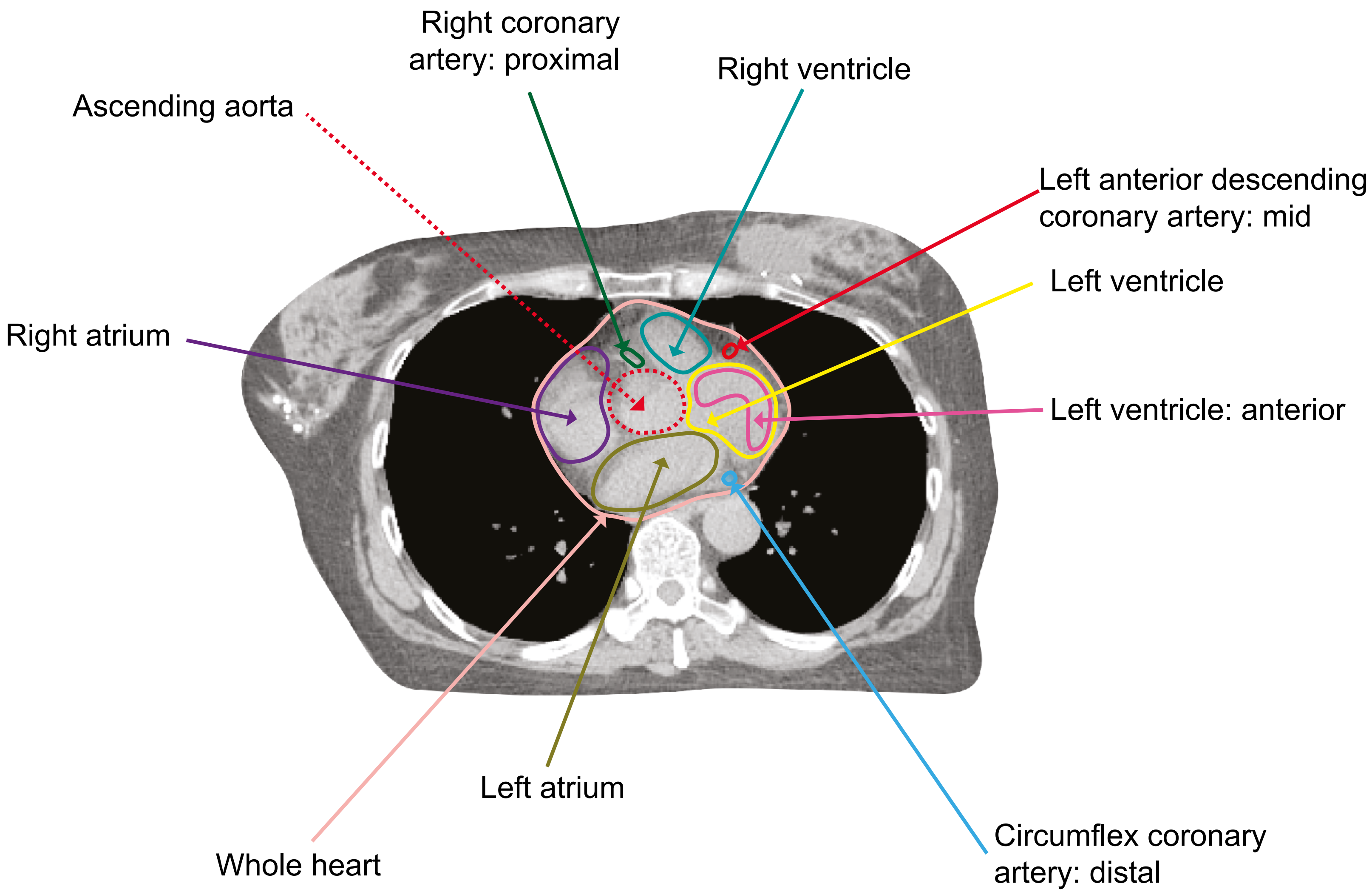
Right coronary artery: proximal
Right ventricle
Ascending aorta
Left anterior descending coronary artery: mid
Left ventricle
Right atrium
Left ventricle: anterior
Left atrium
Whole heart
Circumflex coronary artery: distal

12

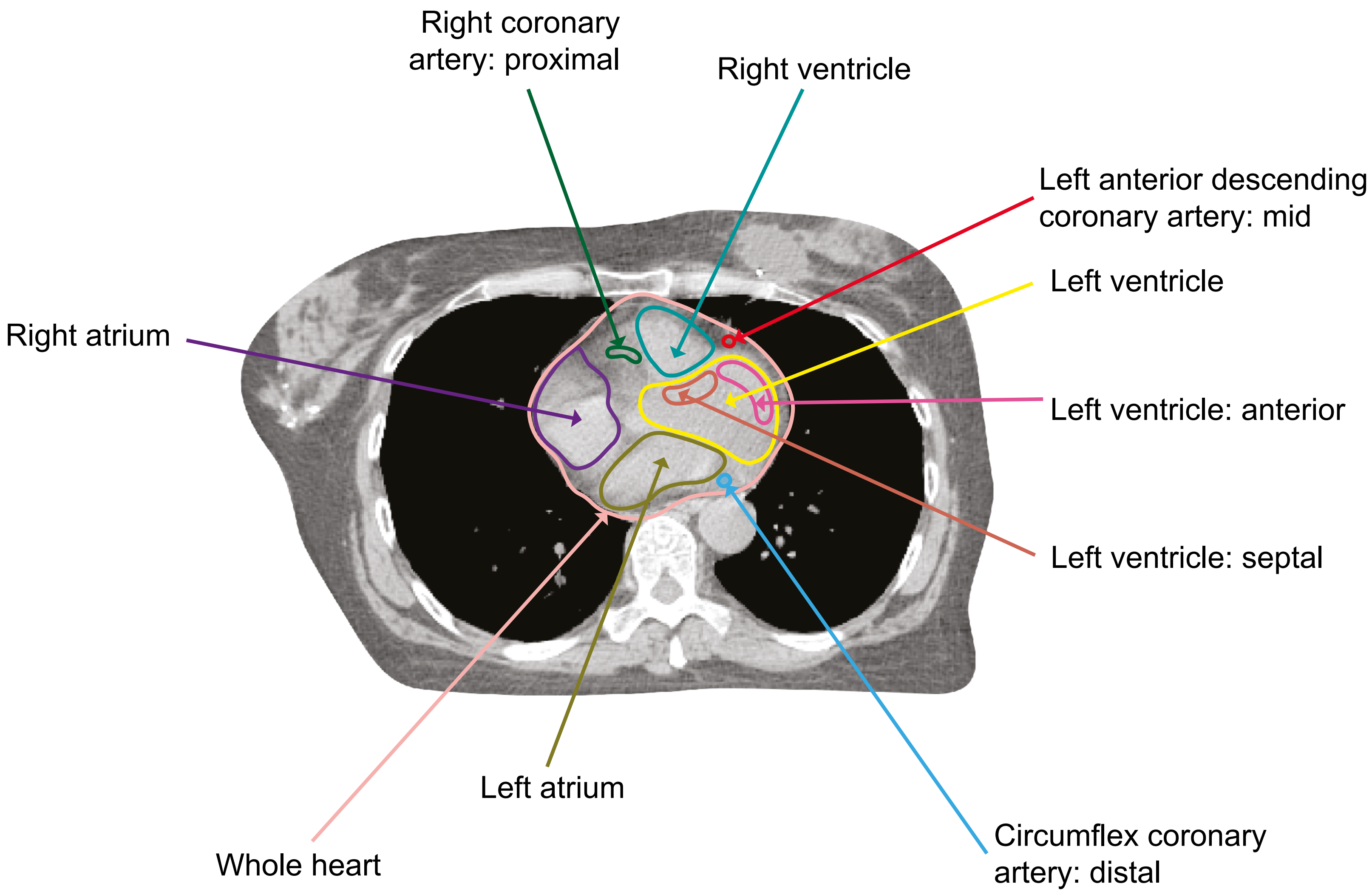
Right coronary artery: proximal
Right ventricle
Left anterior descending coronary artery: mid
Left ventricle
Right atrium
Left ventricle: anterior
Left ventricle: septal
Left atrium
Whole heart
Circumflex coronary artery: distal

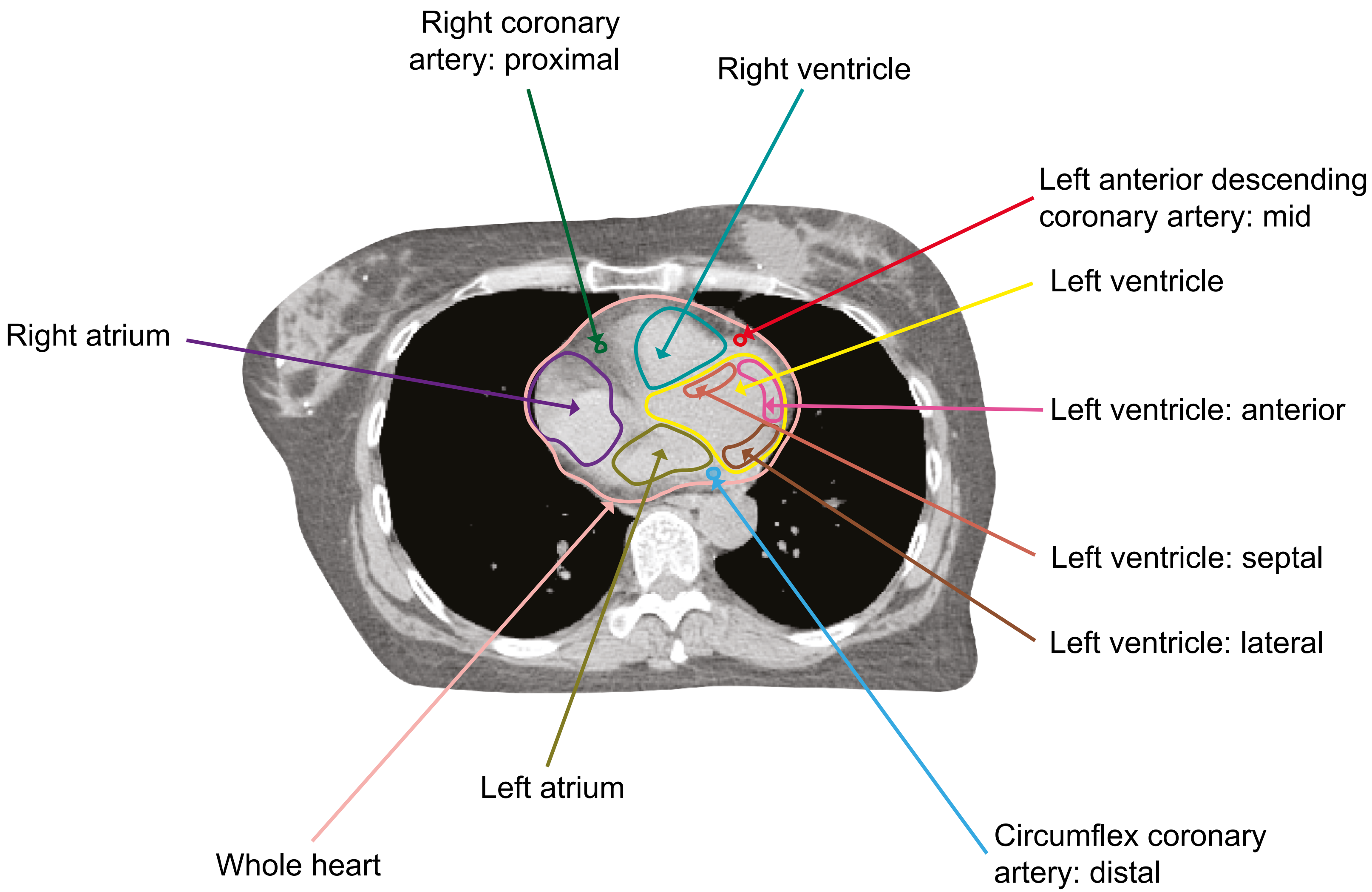
Right coronary
artery: proximal
Right ventricle
Left anterior descending
coronary artery: mid
Left ventricle
Right atrium
Left ventricle: anterior
Left ventricle: septal
Left ventricle: lateral
Left atrium
Whole heart
Circumflex coronary
artery: distal

14

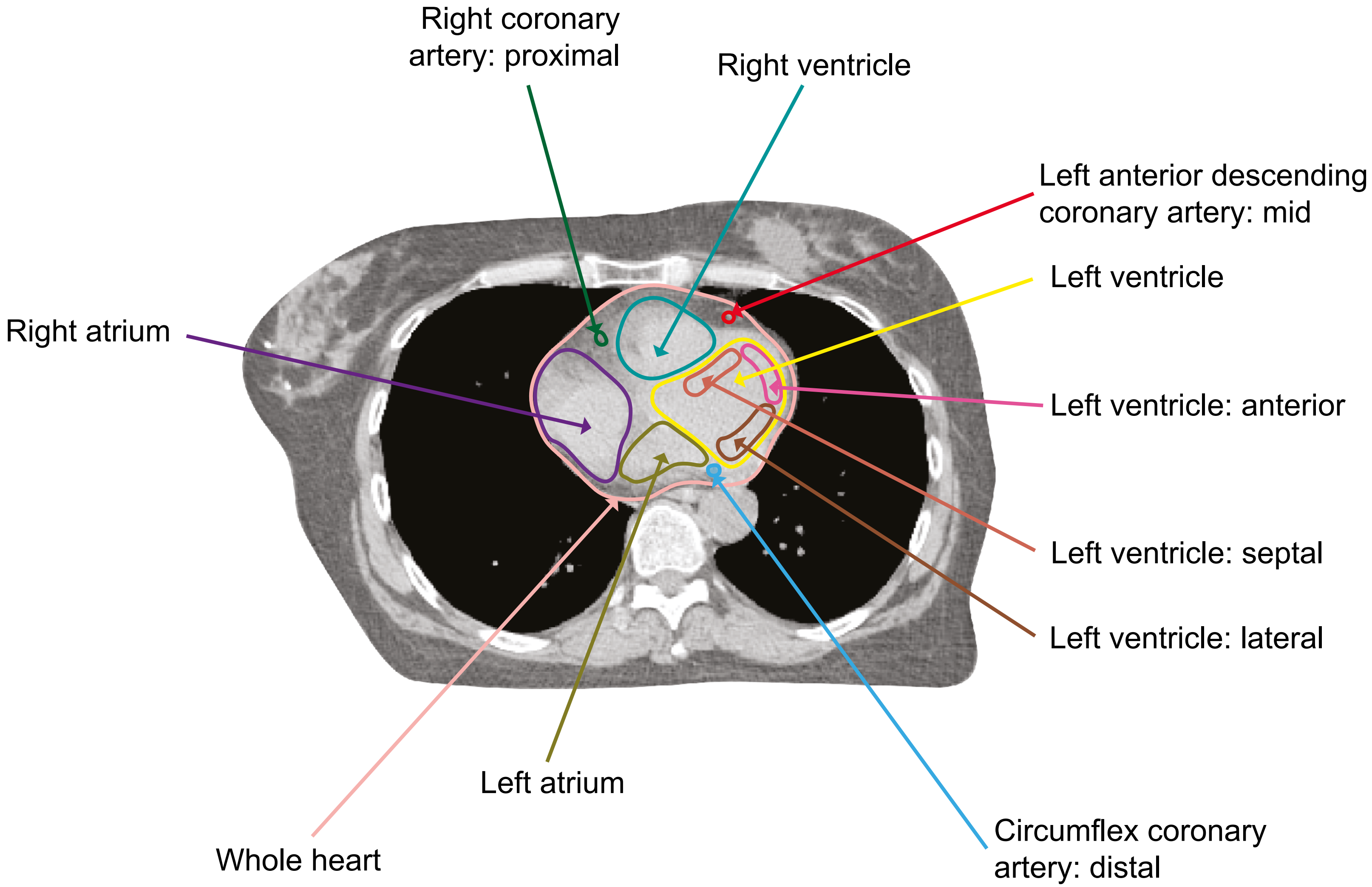
Right coronary artery: proximal
Right ventricle
Left anterior descending coronary artery: mid
Left ventricle
Right atrium
Left ventricle: anterior
Left ventricle: septal
Left ventricle: lateral
Left atrium
Whole heart
Circumflex coronary artery: distal

15

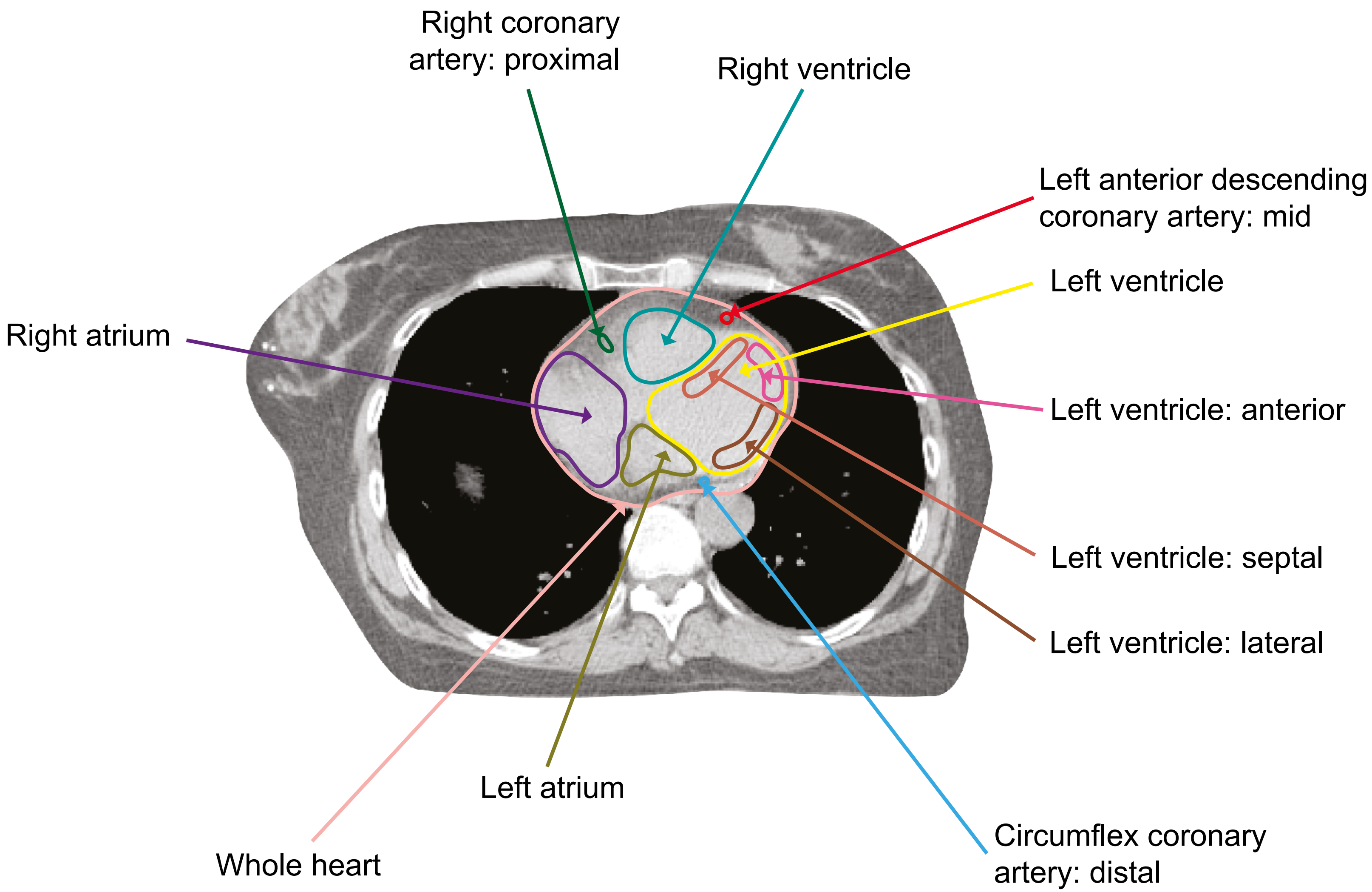
Right coronary artery: proximal
Right ventricle
Left anterior descending coronary artery: mid
Left ventricle
Right atrium
Left ventricle: anterior
Left ventricle: septal
Left ventricle: lateral
Left atrium
Whole heart
Circumflex coronary artery: distal

16

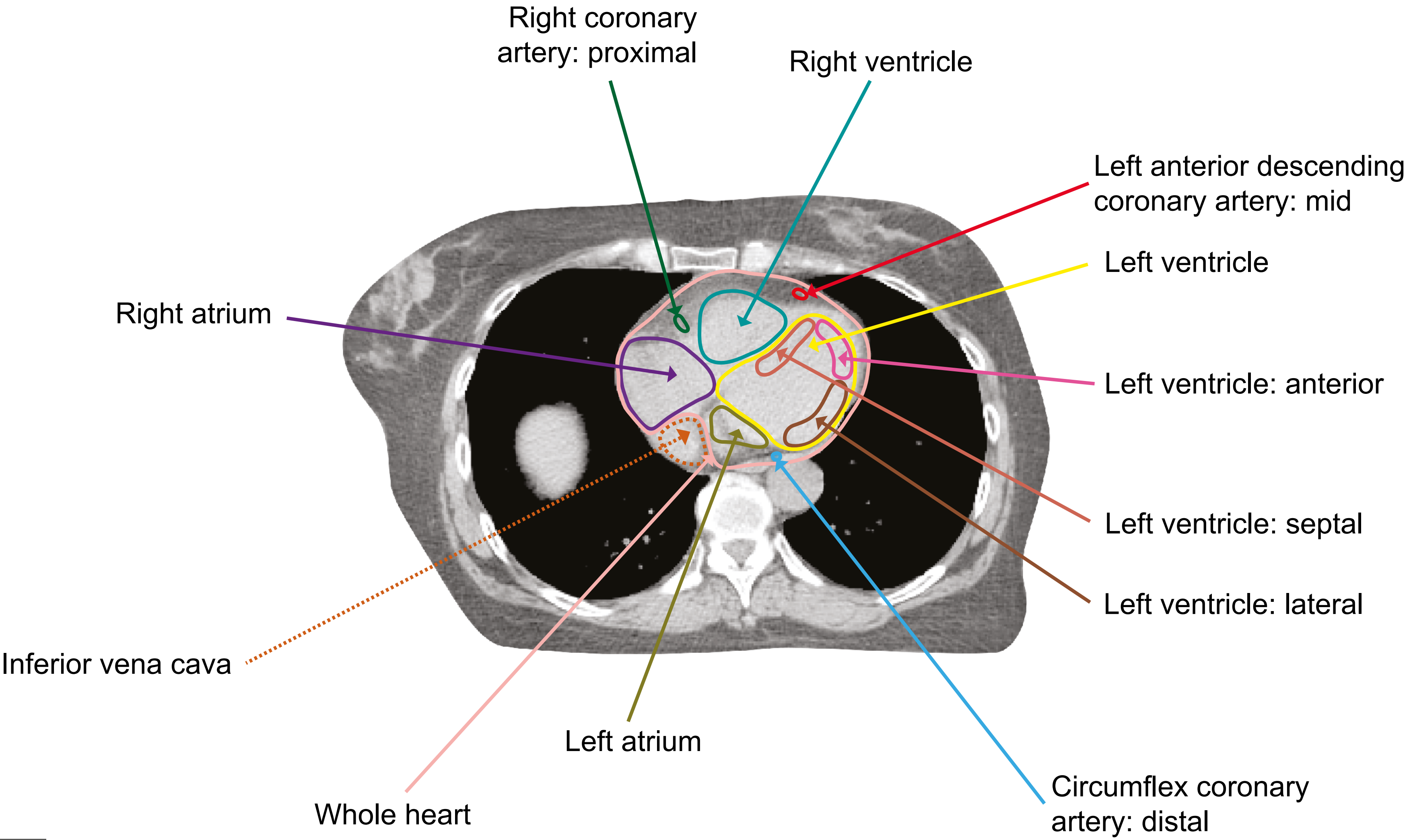
Right coronary artery: proximal
Right ventricle
Left anterior descending coronary artery: mid
Left ventricle
Right atrium
Left ventricle: anterior
Left ventricle: septal
Left ventricle: lateral
Inferior vena cava
Left atrium
Whole heart
Circumflex coronary artery: distal

17

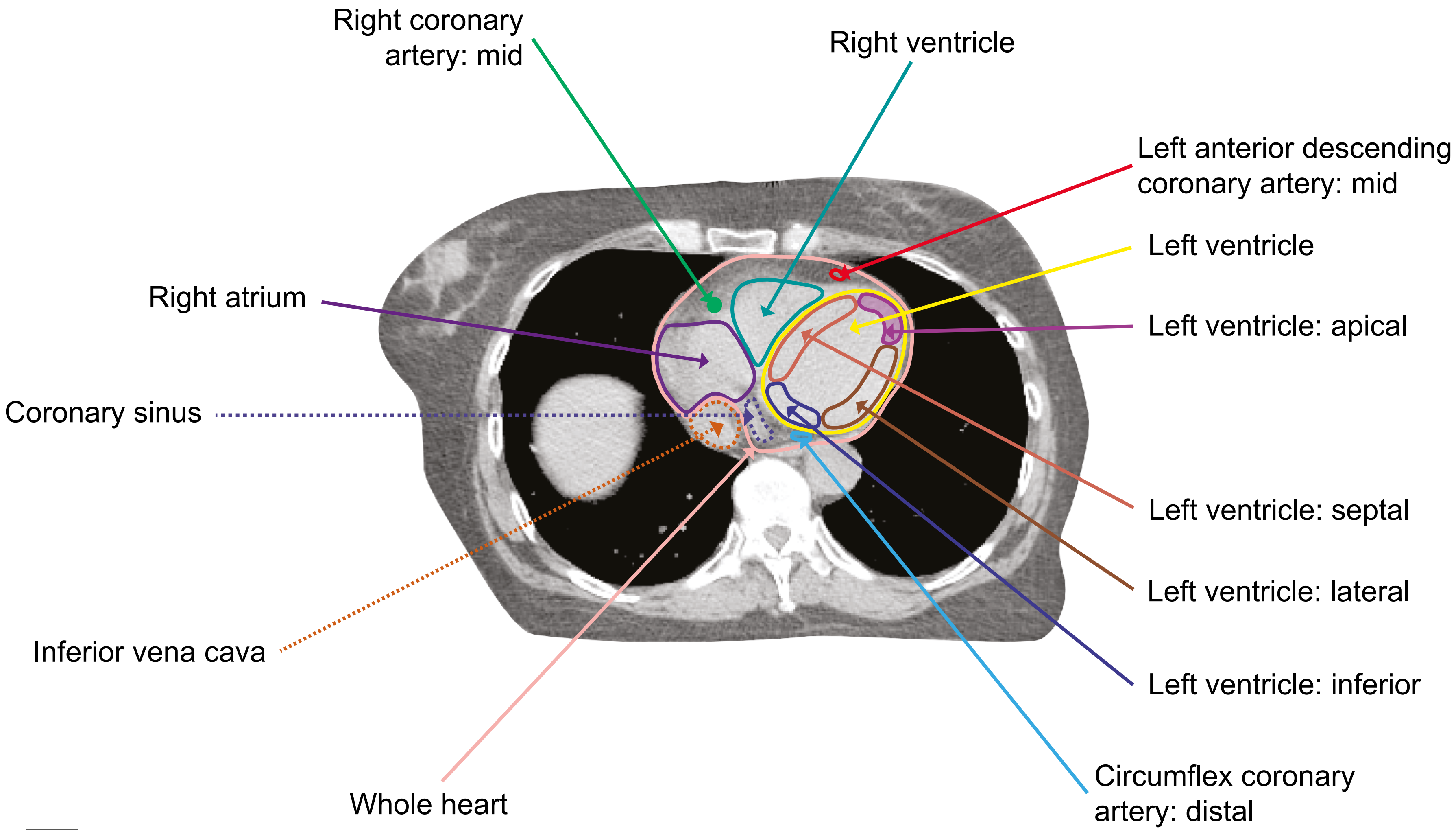
Right coronary artery: mid
Right ventricle
Left anterior descending coronary artery: mid
Left ventricle
Left ventricle: apical
Right atrium
Coronary sinus
Left ventricle: septal
Left ventricle: lateral
Inferior vena cava
Left ventricle: inferior
Whole heart
Circumflex coronary artery: distal

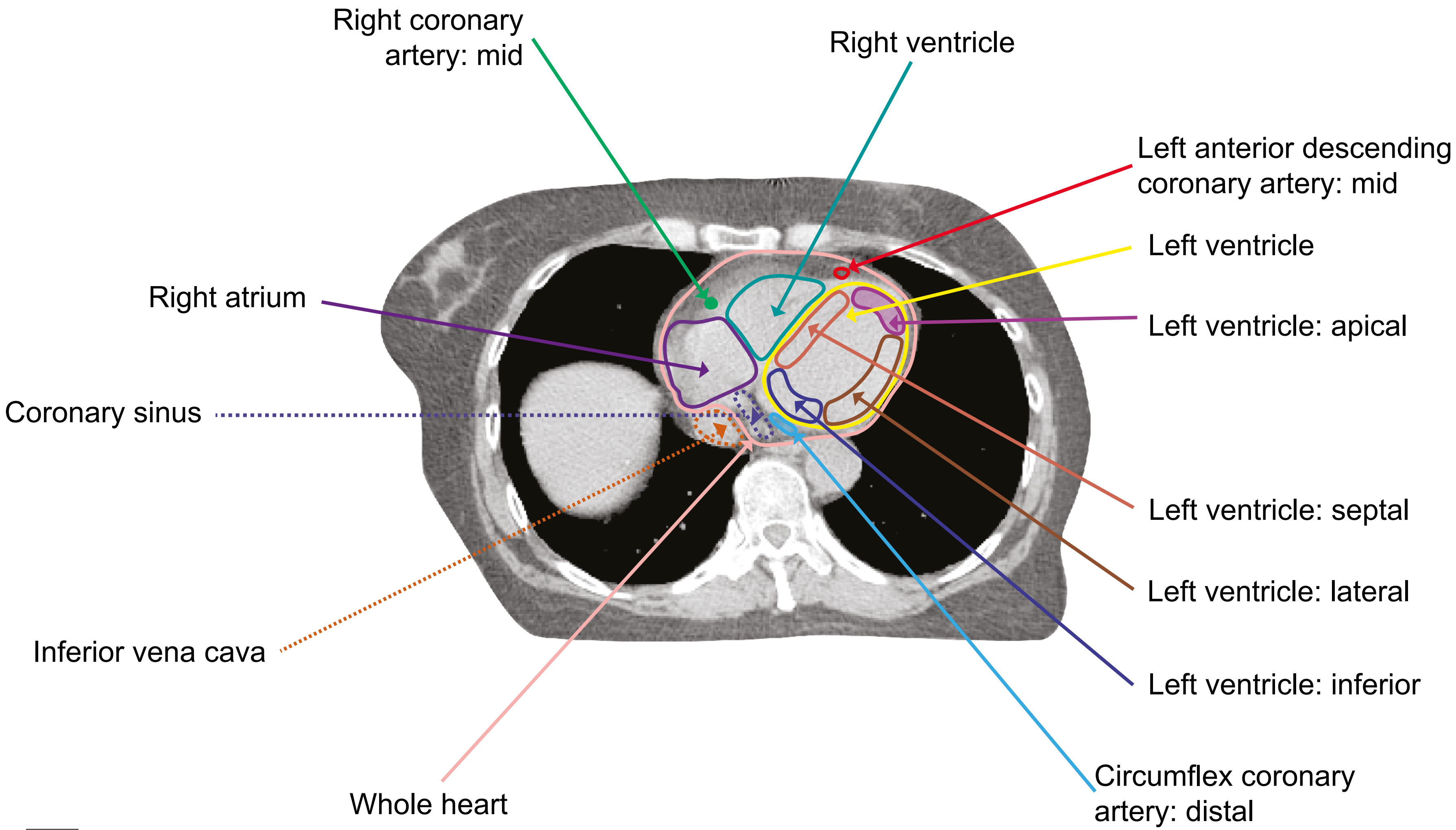
Right coronary artery: mid
Right ventricle
Left anterior descending coronary artery: mid
Left ventricle
Right atrium
Left ventricle: apical
Coronary sinus
Left ventricle: septal
Left ventricle: lateral
Inferior vena cava
Left ventricle: inferior
Whole heart
Circumflex coronary artery: distal

19

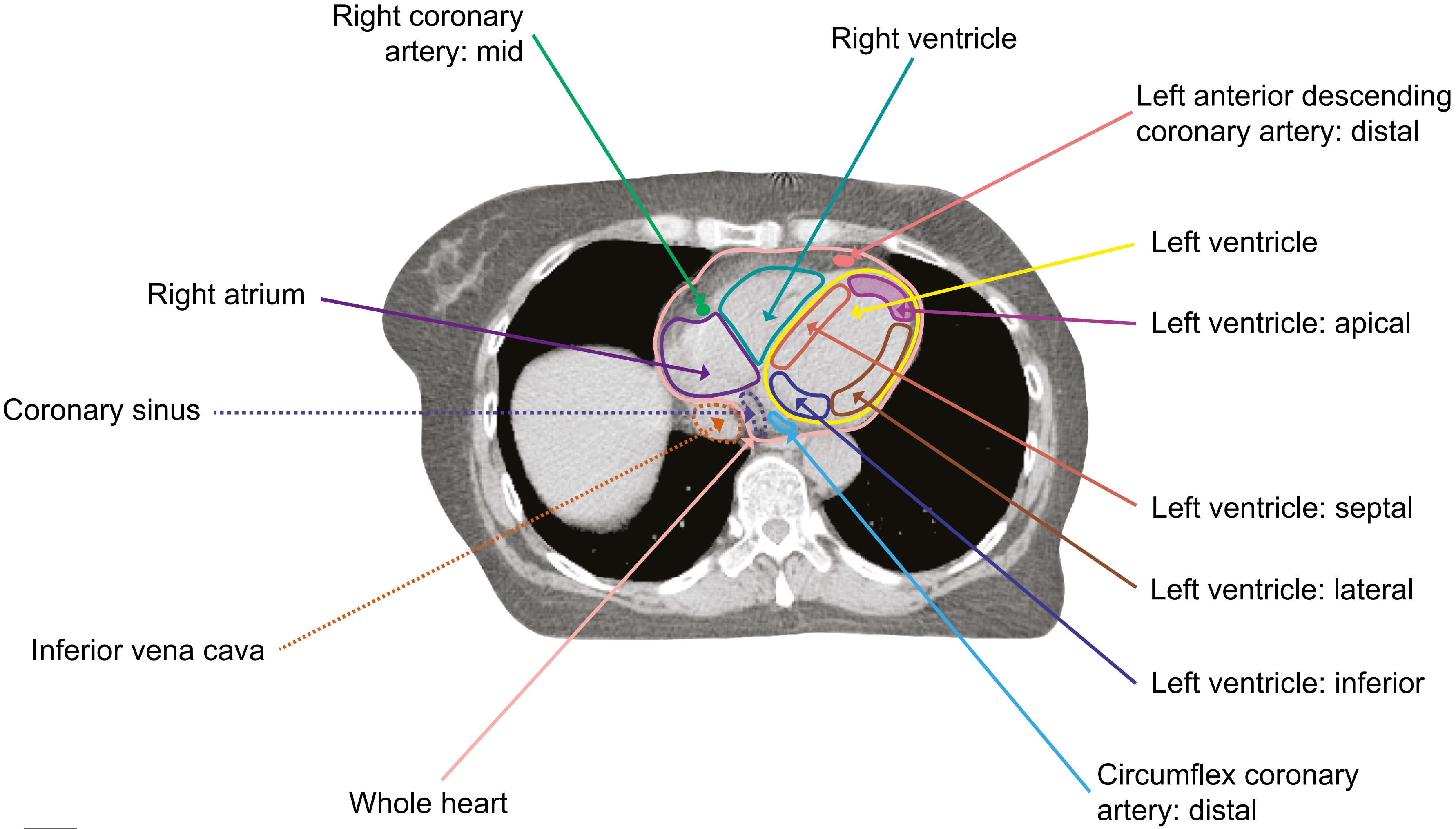

Right coronary artery: mid
Right ventricle
Left anterior descending coronary artery: distal
Left ventricle
Left ventricle: apical
Right atrium
Coronary sinus
Left ventricle: septal
Left ventricle: lateral
Inferior vena cava
Left ventricle: inferior
Circumflex coronary artery: distal
Whole heart

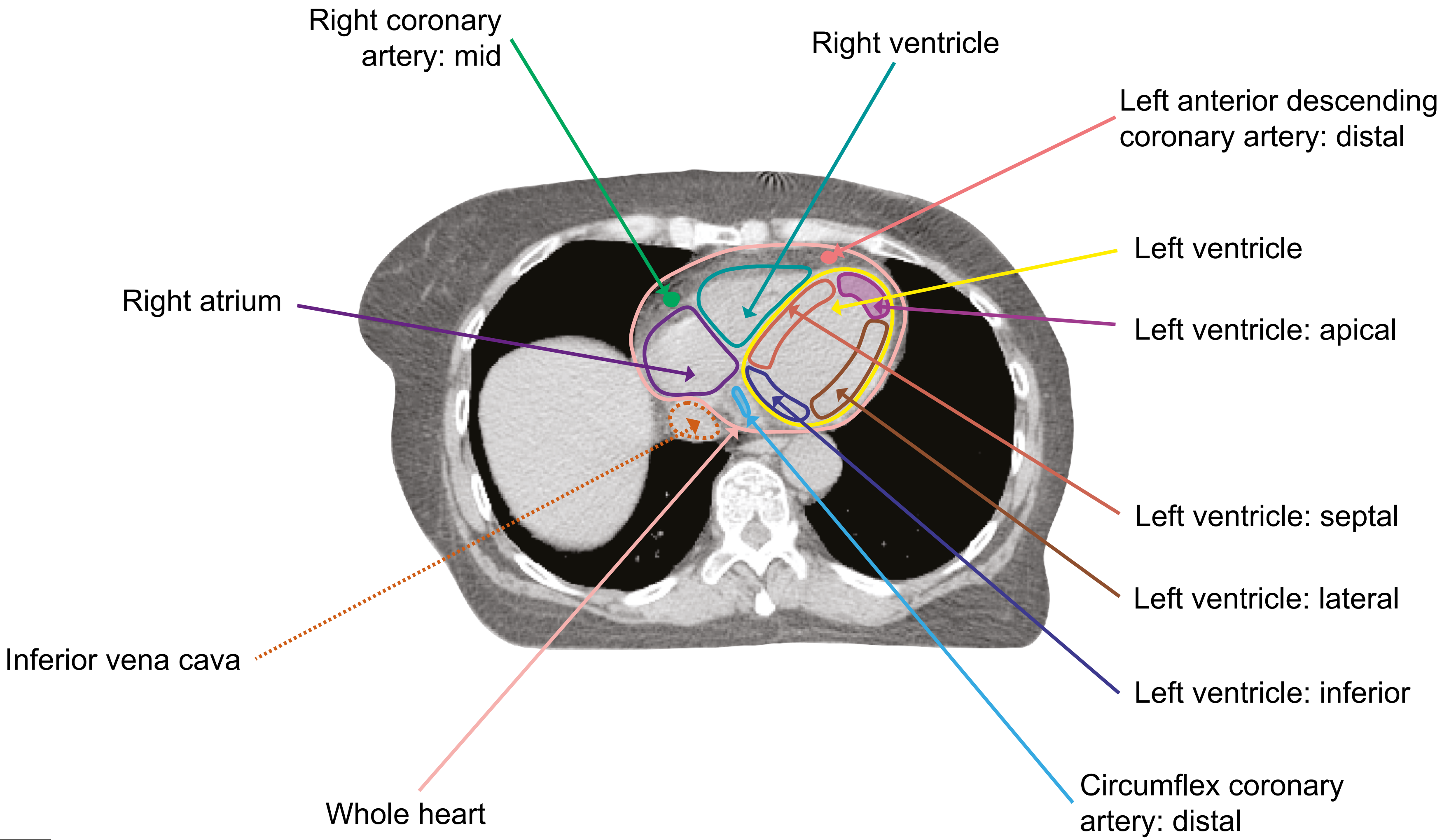
Right coronary artery: mid
Right ventricle
Left anterior descending coronary artery: distal
Left ventricle
Right atrium
Left ventricle: apical
Left ventricle: septal
Left ventricle: lateral
Inferior vena cava
Left ventricle: inferior
Circumflex coronary artery: distal
Whole heart

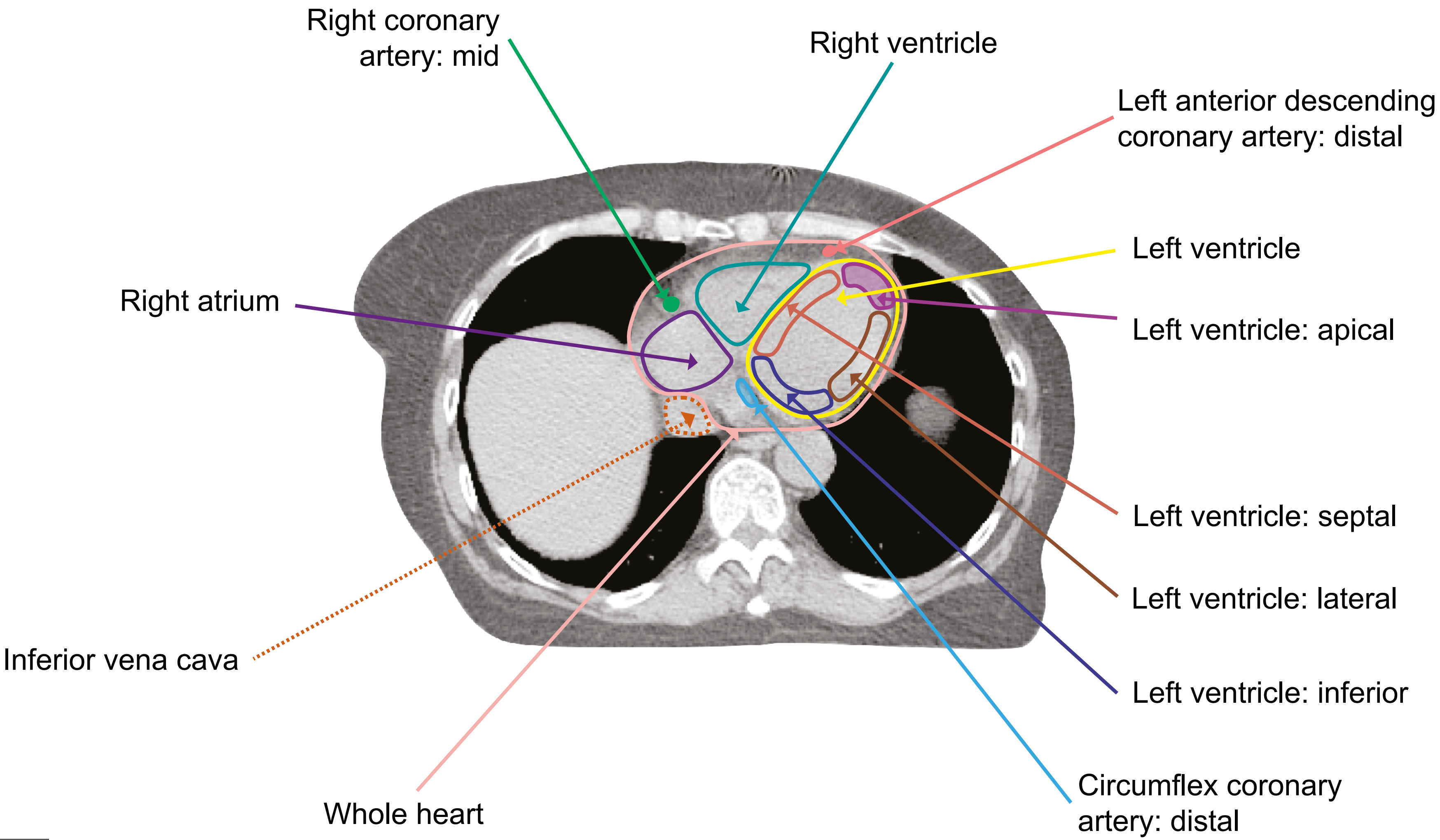
Right coronary artery: mid
Right ventricle
Left anterior descending coronary artery: distal
Left ventricle
Right atrium
Left ventricle: apical
Left ventricle: septal
Left ventricle: lateral
Inferior vena cava
Left ventricle: inferior
Whole heart
Circumflex coronary artery: distal

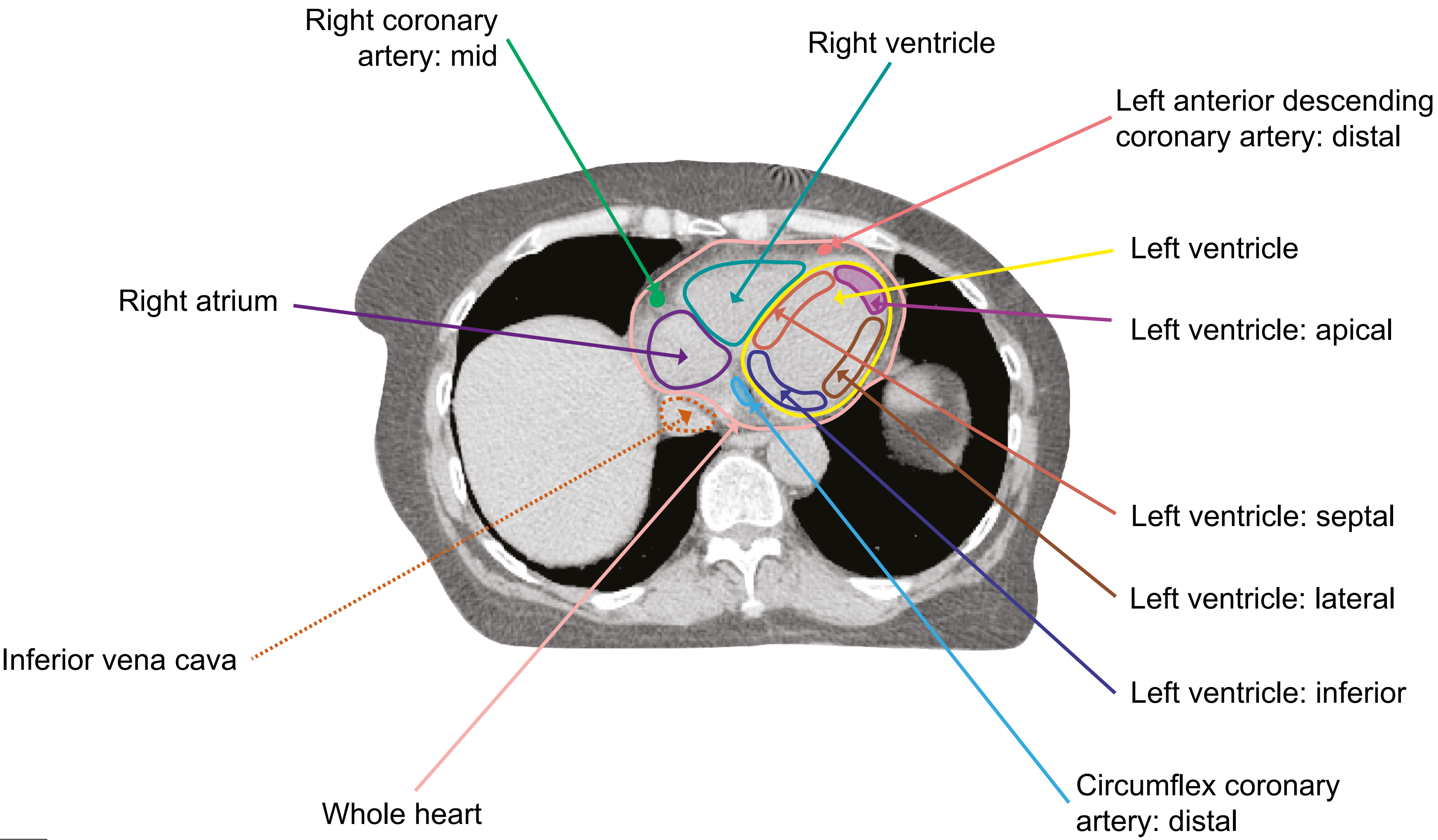
Right coronary artery: mid
Right ventricle
Left anterior descending coronary artery: distal
Left ventricle
Right atrium
Left ventricle: apical
Left ventricle: septal
Left ventricle: lateral
Inferior vena cava
Left ventricle: inferior
Whole heart
Circumflex coronary artery: distal

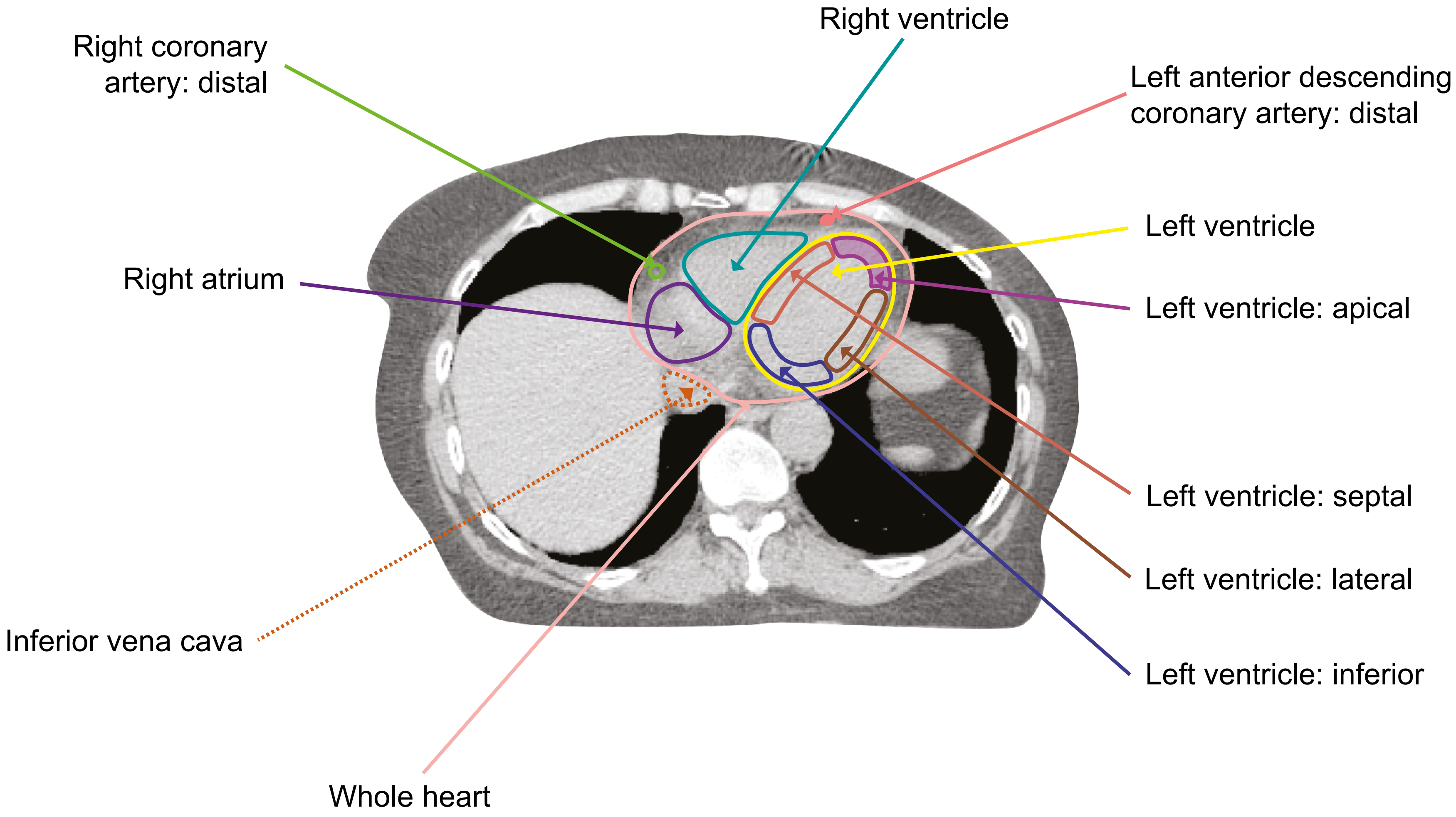
Right coronary artery: distal
Right ventricle
Left anterior descending coronary artery: distal
Left ventricle
Right atrium
Left ventricle: apical
Left ventricle: septal
Left ventricle: lateral
Inferior vena cava
Left ventricle: inferior
Whole heart

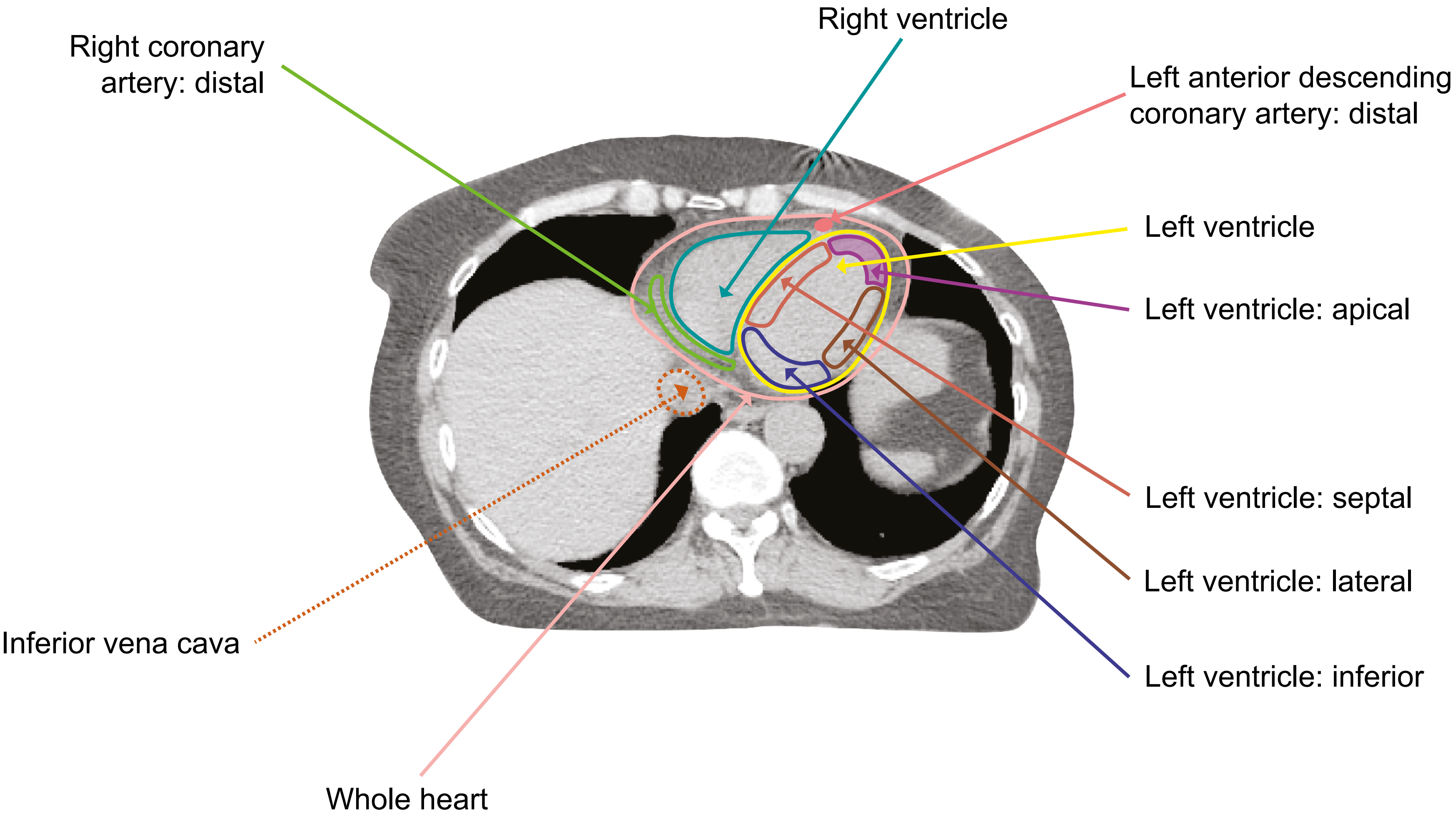
Right coronary artery: distal
Right ventricle
Left anterior descending coronary artery: distal
Left ventricle
Left ventricle: apical
Left ventricle: septal
Left ventricle: lateral
Left ventricle: inferior
Inferior vena cava
Whole heart

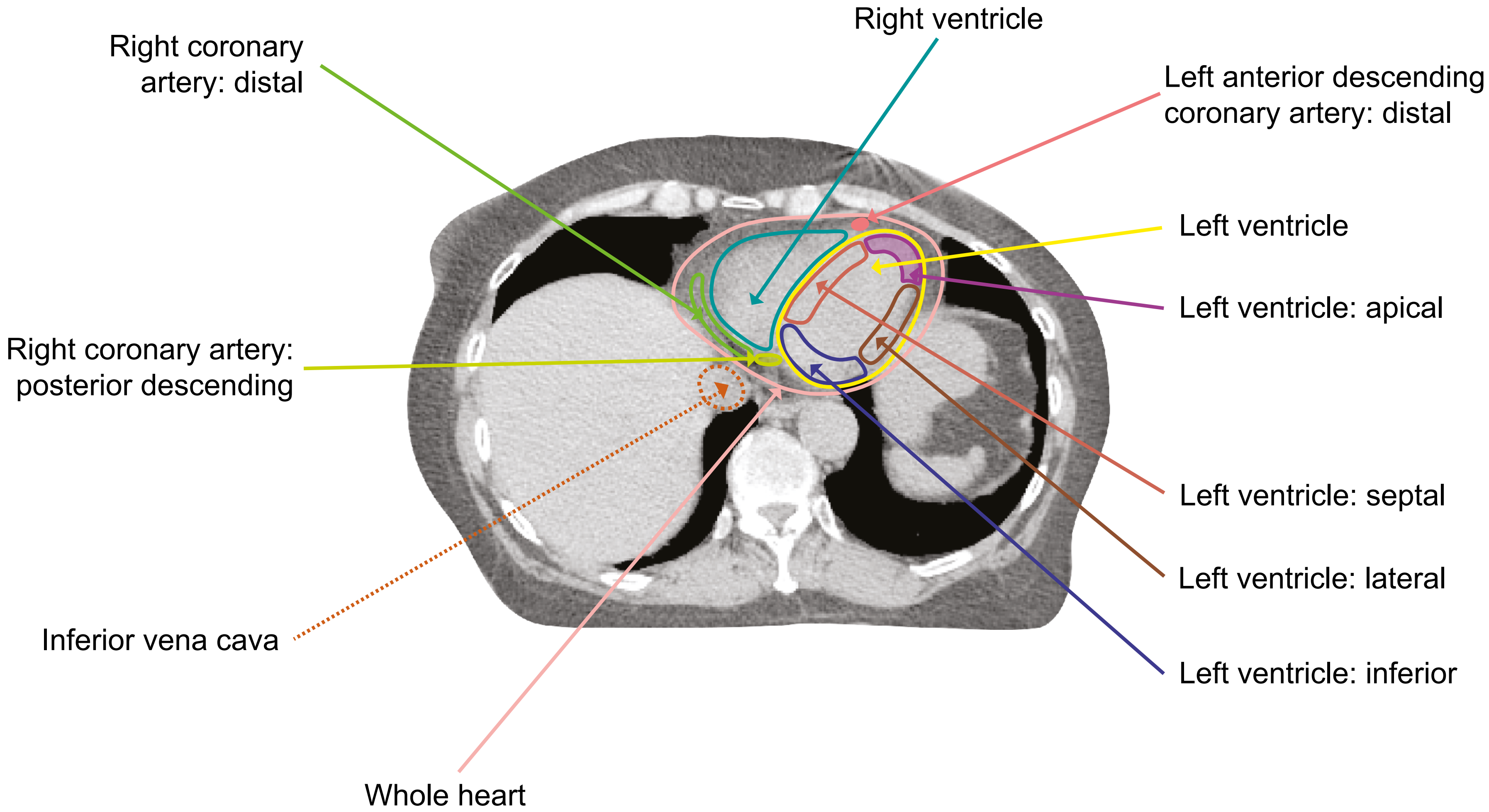
Right ventricle
Right coronary artery: distal
Left anterior descending coronary artery: distal
Left ventricle
Left ventricle: apical
Right coronary artery: posterior descending
Left ventricle: septal
Left ventricle: lateral
Inferior vena cava
Left ventricle: inferior
Whole heart

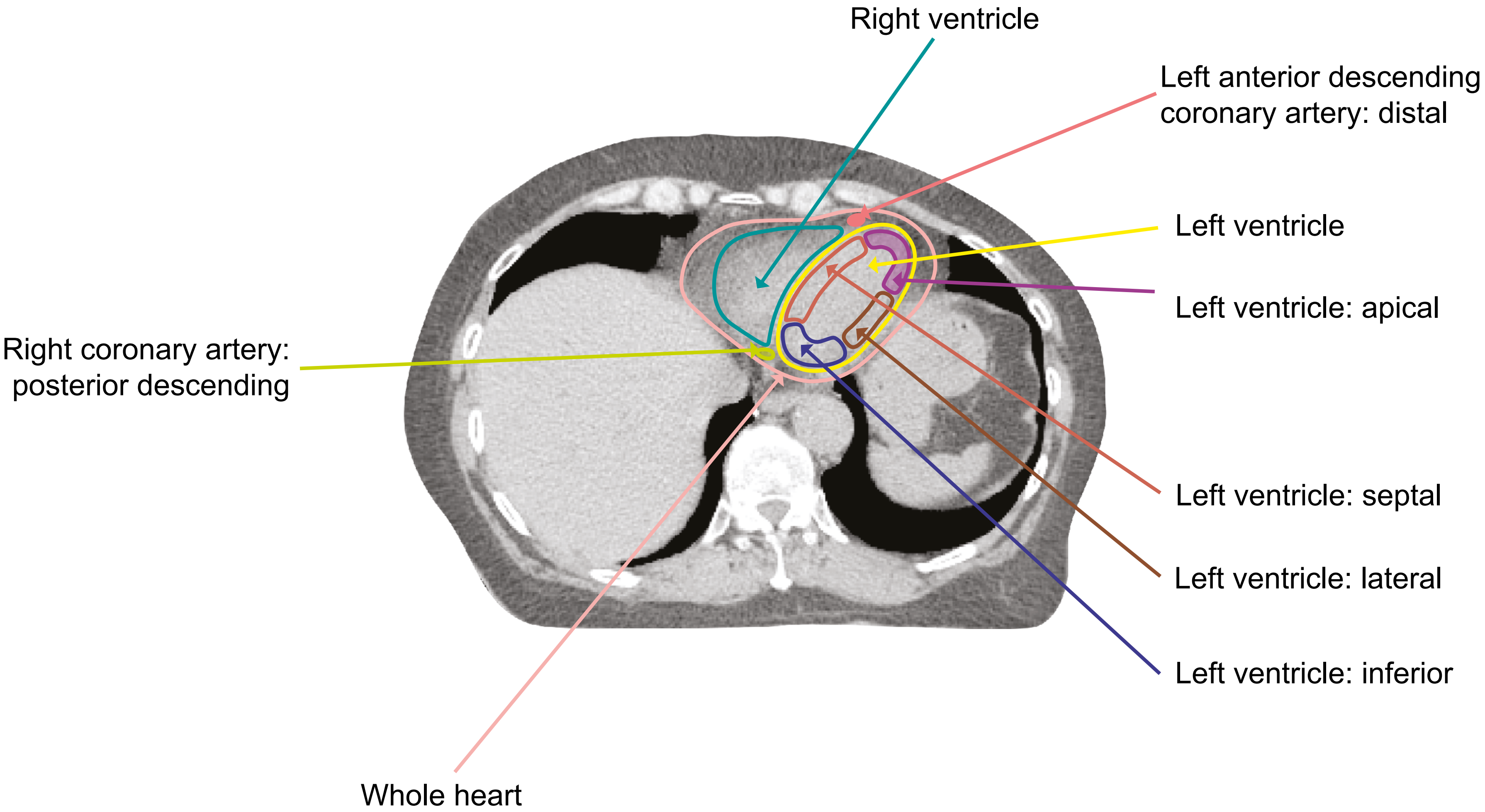
Right ventricle
Left anterior descending coronary artery: distal
Left ventricle
Left ventricle: apical
Right coronary artery: posterior descending
Left ventricle: septal
Left ventricle: lateral
Left ventricle: inferior
Whole heart

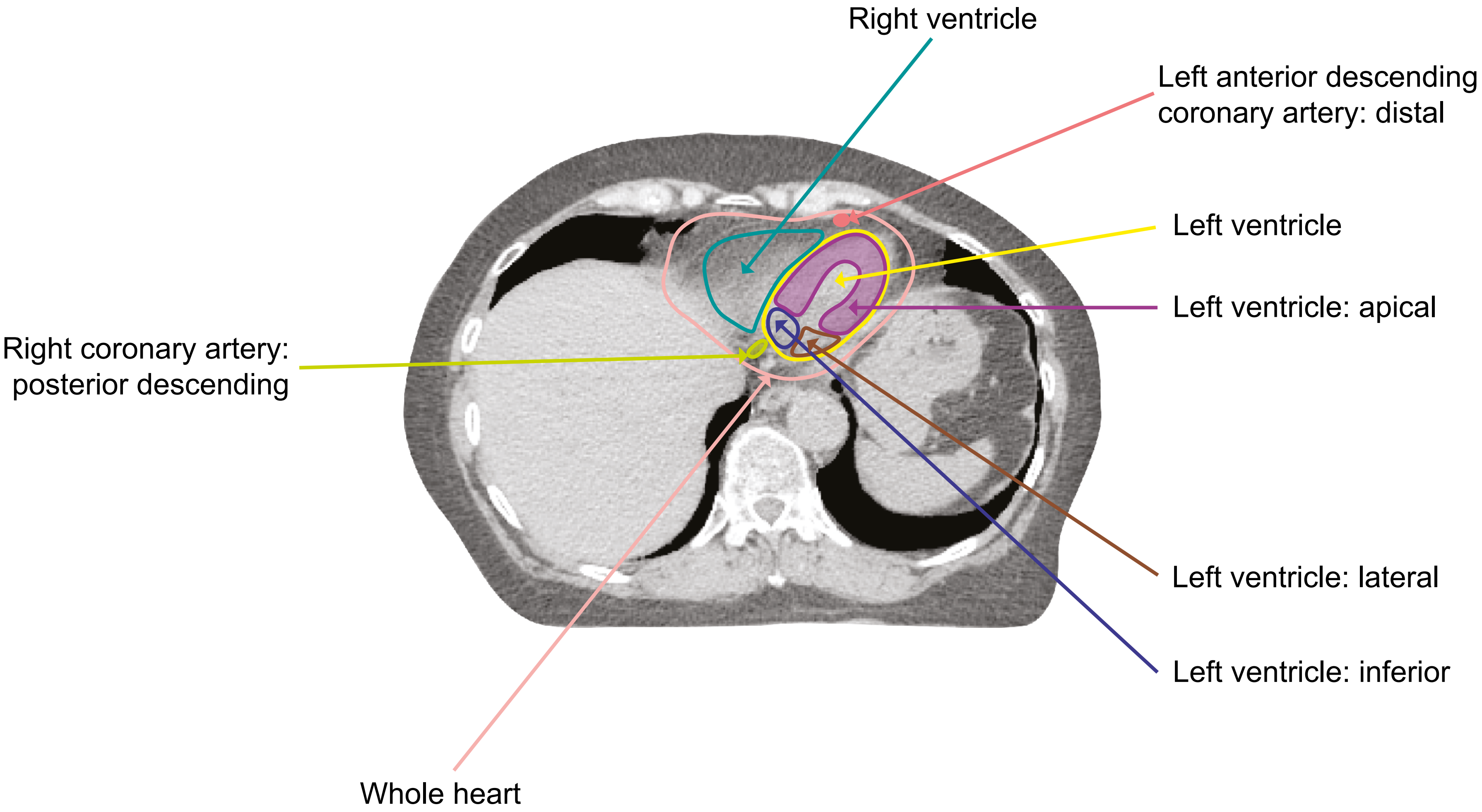
Right ventricle
Left anterior descending coronary artery: distal
Left ventricle
Left ventricle: apical
Right coronary artery: posterior descending
Left ventricle: lateral
Left ventricle: inferior
Whole heart

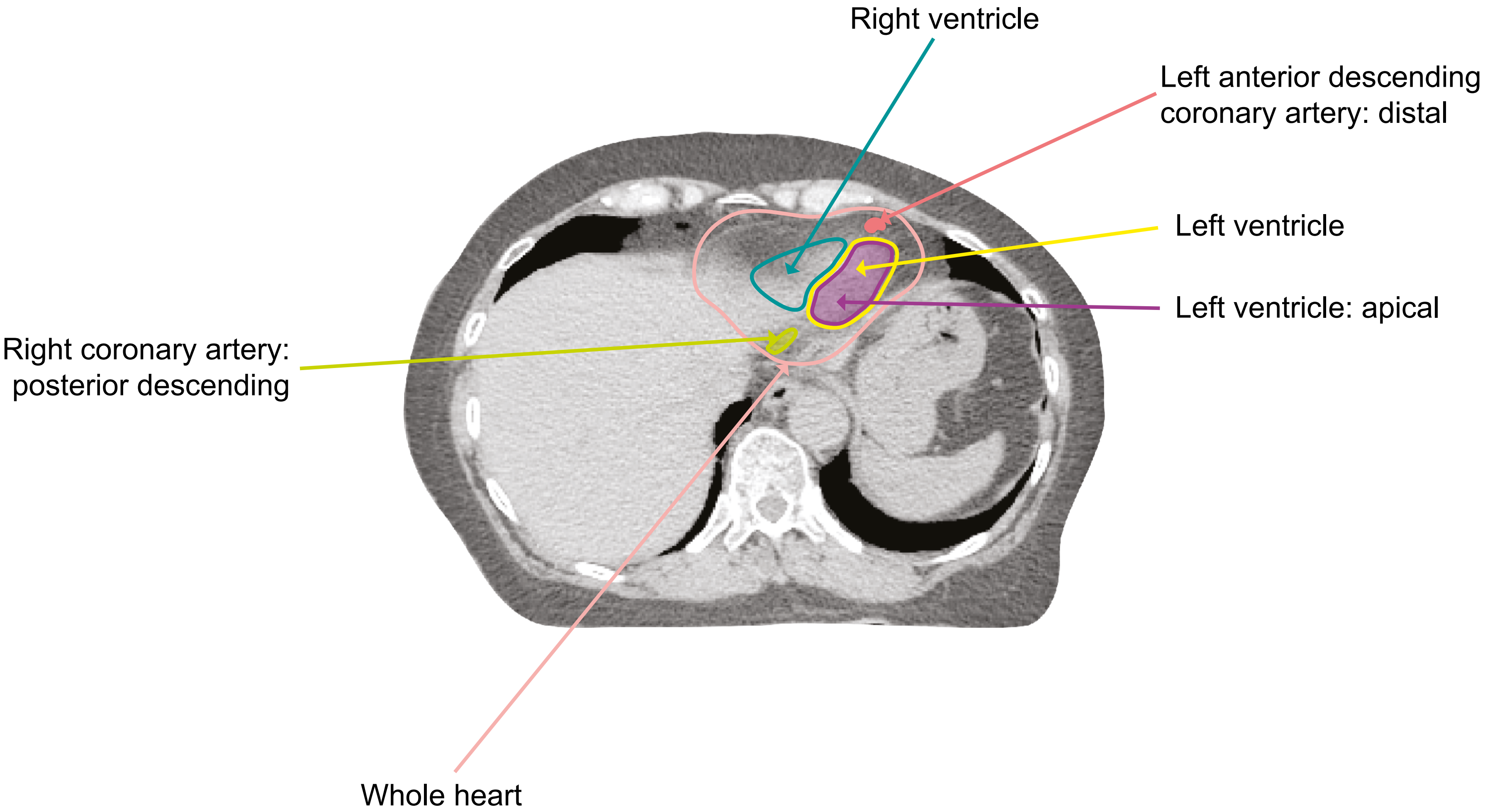
Right ventricle
Left anterior descending coronary artery: distal
Left ventricle
Left ventricle: apical
Right coronary artery: posterior descending
Whole heart

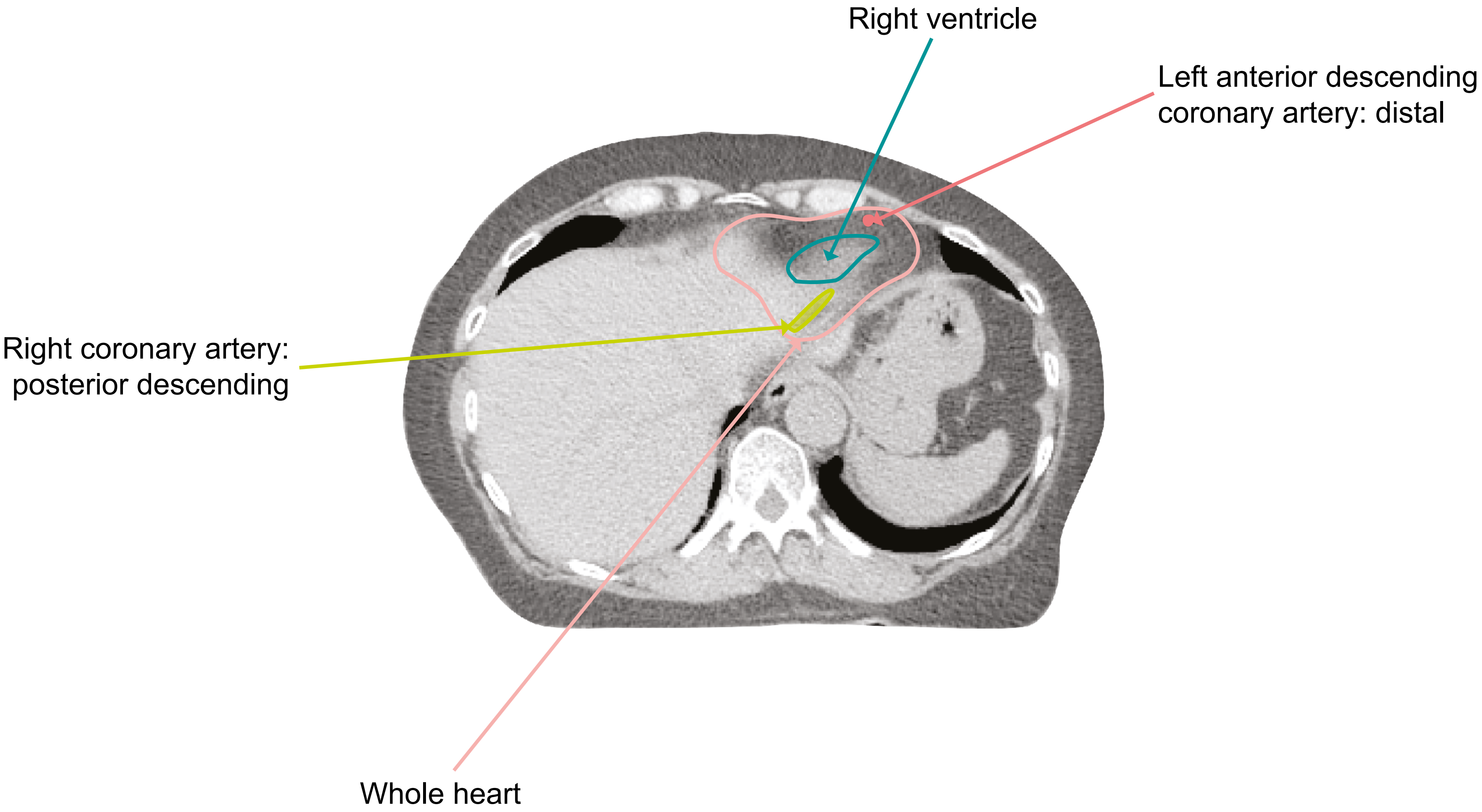
Right ventricle
Left anterior descending coronary artery: distal
Right coronary artery: posterior descending
Whole heart

30

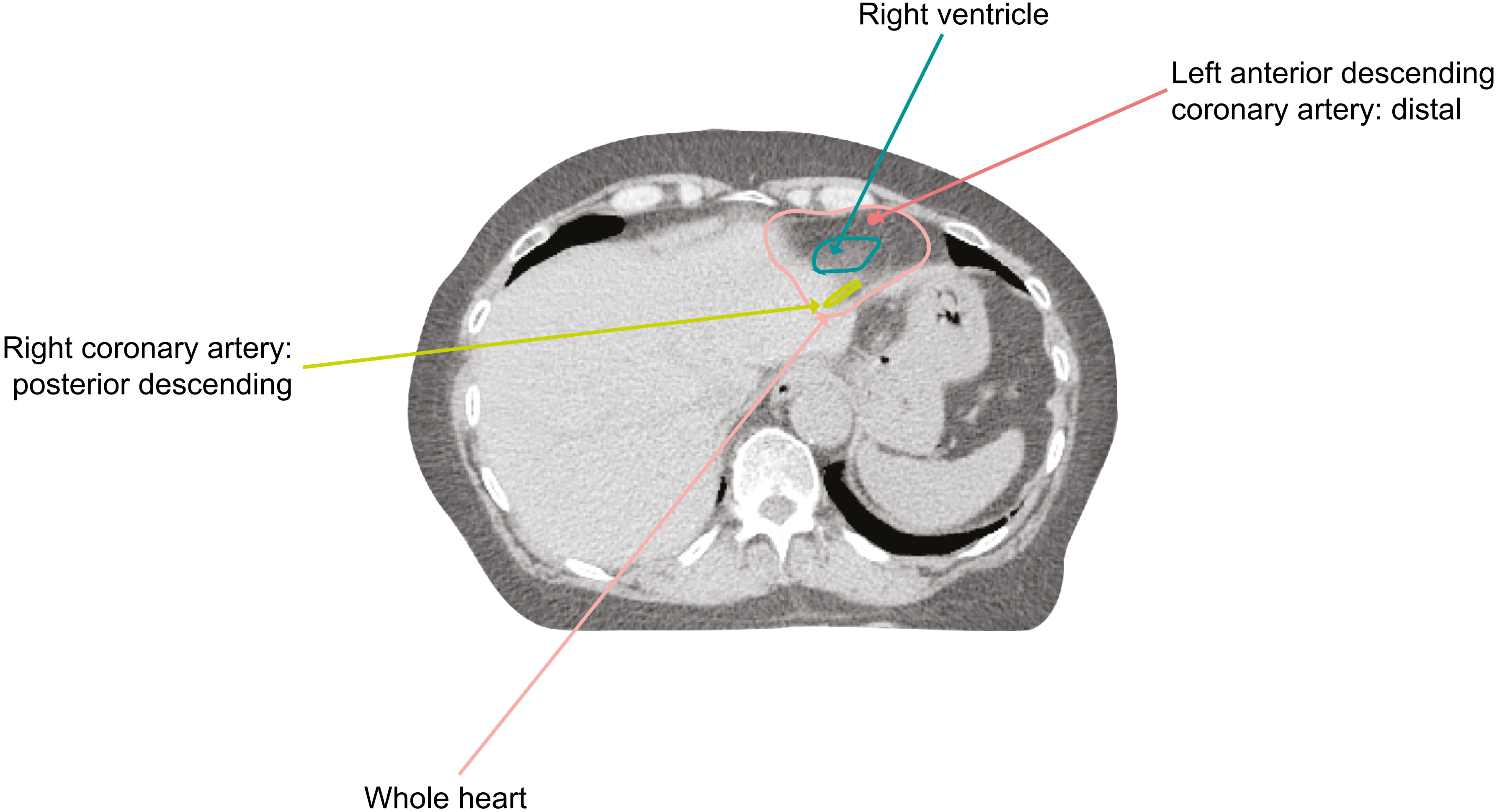
Right ventricle
Left anterior descending coronary artery: distal
Right coronary artery: posterior descending
Whole heart

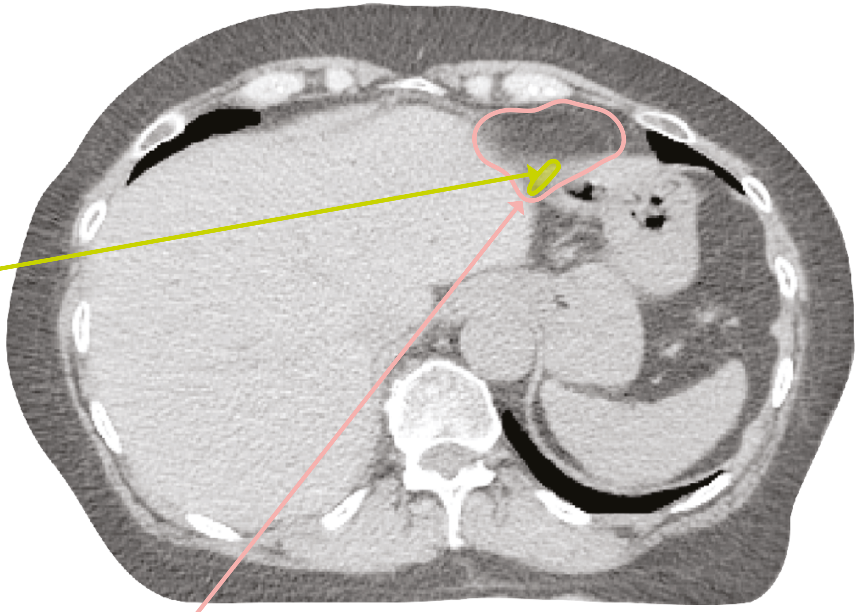
Right coronary artery: posterior descending
Whole heart

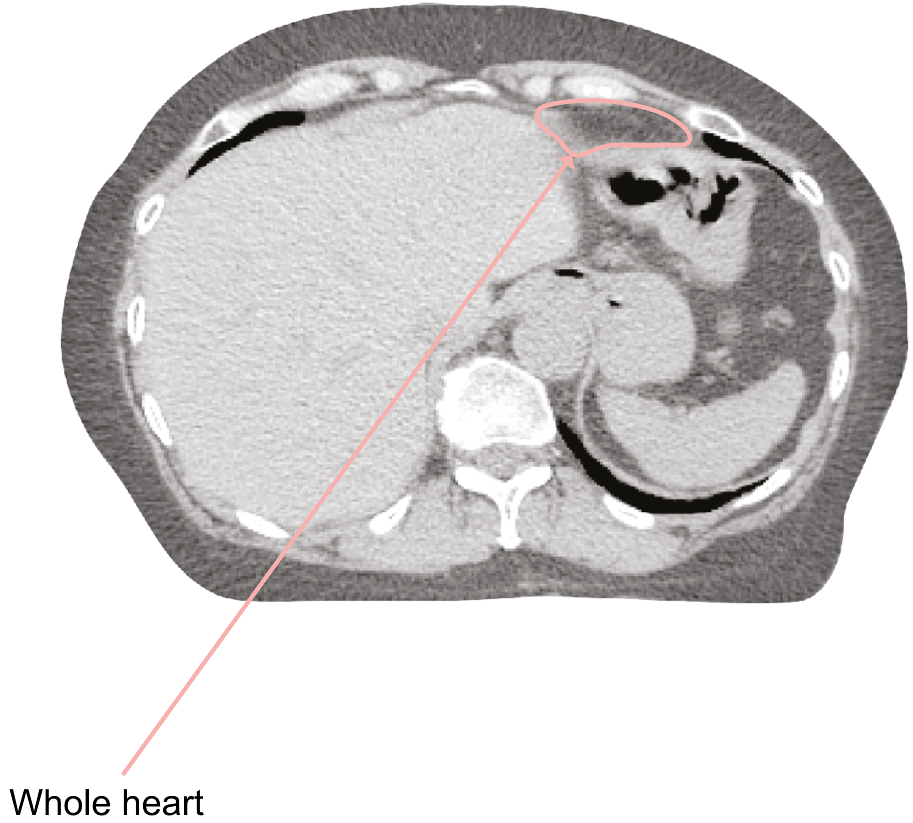

33

Supplementary Fig. 4. Cardiac substructure contours by six observers on one CT-planning scan at the level of the left ventricle superiorly (a) and inferiorly (b).

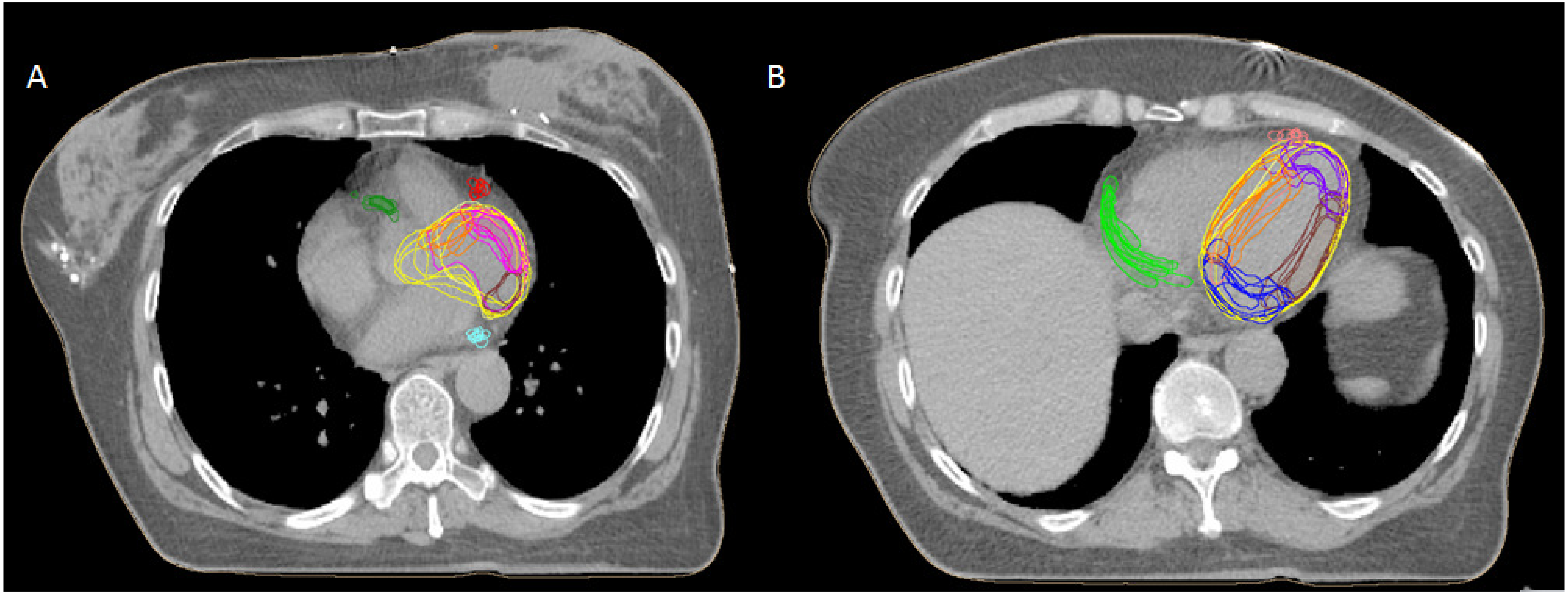

Supplementary Table 1. Variation in doses from right breast cancer regimens for cardiac segments contoured by six observers on one CT-planning dataset.

Cardiac segments	Dose variation (Gy), right-sided regimens Mean (SD), range		
	PWT	OPP	E⁻/MV
Left ventricle	0.3 (0.0), 0.3-0.3	0.2 (0.0), 0.2-0.2	0.7 (0.0), 0.7-0.7
LV: apical	0.2 (0.0), 0.2-0.2	0.1 (0.0), 0.1-0.1	0.5 (0.0), 0.5-0.5
LV: lateral	0.2 (0.0), 0.2-0.2	0.1 (0.0), 0.1-0.1	0.5 (0.0), 0.5-0.5
LV: inferior	0.3 (0.1), 0.3-0.4	0.3 (0.1), 0.2-0.3	0.9 (0.1), 0.8-1.0
LV: septal	0.5 (0.0), 0.4-0.5	0.4 (0.0), 0.4-0.5	0.9 (0.1), 0.8-1.0
LV: anterior	0.3 (0.0), 0.3-0.4	0.3 (0.0), 0.2-0.3	0.9 (0.1), 0.8-1.0
LMCA	0.8 (0.1), 0.7-6.8	0.8 (0.0), 0.8-0.8	1.9 (0.1), 1.8-2.0
LADCA proximal	0.6 (0.0),0.6-0.6	0.6 (0.1), 0.5-0.6	1.1 (0.1),1.0-1.2
LADCA mid	0.5 (0.0), 0.5-0.5	0.4 (0.0), 0.4-0.4	0.8 (0.0), 0.7-0.8
LADCA distal	0.2 (0.1), 0.1-0.3	0.1 (0.1), 0.1-0.2	0.6 (0.1), 0.5-0.6
Cx proximal	0.6 (0.0), 0.6-0.7	0.6 (0.1), 0.5-0.7	1.4 (0.1), 1.2-1.6
Cx distal	0.3 (0.0), 0.3-0.4	0.2 (0.1), 0.1-0.3	0.9 (0.1), 0.8-1.0
RCA proximal	2.3 (0.2), 1.9-2.5	2.5 (0.3), 2.1-2.8	**16.9 (3.6), 12.6-21.7**
RCA mid	**3.1 (1.3), 2.2-5.7**	**4.2 (2.5), 2.8-9.2**	**28.6 (3.7), 25.6-35.6**
RCA distal	1.4 (0.3), 1.0-1.8	1.7 (0.40), 1.2-2.1	**20.3(2.2), 18.1-23.6**
RCA post desc	0.4 (0.0),0.3-0.4	0.4 (0.1), 0.3-0.5	1.4 (0.4), 1.0-2.2

Dose variation results for structures located near the high dose-gradient are highlighted in bold.

Abbreviations: PWT: partially wide tangential technique; OPP: oblique parasternal photon technique; E⁻/MV: mixed electron/photon direct parasternal technique; LV: left ventricle; LMCA: left main coronary artery, LADCA: left anterior descending coronary artery, Cx: circumflex coronary artery, RCA: right coronary artery, post desc: posterior descending
